# Fine-tuning of amino sugar homeostasis by EIIA^Ntr^ in *Salmonella* Typhimurium

**DOI:** 10.1038/srep33055

**Published:** 2016-09-15

**Authors:** Woongjae Yoo, Hyunjin Yoon, Yeong-Jae Seok, Chang-Ro Lee, Hyung Ho Lee, Sangryeol Ryu

**Affiliations:** 1 Department of Food and Animal Biotechnology, Department of Agricultural Biotechnology, Research Institute for Agriculture and Life Sciences, and Center for Food and Bioconvergence, Seoul National University, Seoul 08826, Korea; 2 Department of Molecular Science and Technology, Department of Applied Chemistry and Biological Engineering, Ajou University, Suwon 16499, Korea; 3 Department of Biological Sciences and Institute of Microbiology, Seoul National University, Seoul 08826, Korea; 4 Department of Biological Sciences, Myongji University, Yongin, Gyeonggido 17058, Republic of Korea; 5 Department of Chemistry, College of Natural Sciences, Seoul National University, Seoul 08826, Korea

## Abstract

The nitrogen-metabolic phosphotransferase system, PTS^Ntr^, consists of the enzymes I^Ntr^, NPr and IIA^Ntr^ that are encoded by *ptsP*, *ptsO*, and *ptsN*, respectively. Due to the proximity of *ptsO* and *ptsN* to *rpoN*, the PTS^Ntr^ system has been postulated to be closely related with nitrogen metabolism. To define the correlation between PTS^Ntr^ and nitrogen metabolism, we performed ligand fishing with EIIA^Ntr^ as a bait and revealed that D-glucosamine-6-phosphate synthase (GlmS) directly interacted with EIIA^Ntr^. GlmS, which converts D-fructose-6-phosphate (Fru6P) into D-glucosamine-6-phosphate (GlcN6P), is a key enzyme producing amino sugars through glutamine hydrolysis. Amino sugar is an essential structural building block for bacterial peptidoglycan and LPS. We further verified that EIIA^Ntr^ inhibited GlmS activity by direct interaction in a phosphorylation-state-dependent manner. EIIA^Ntr^ was dephosphorylated in response to excessive nitrogen sources and was rapidly degraded by Lon protease upon amino sugar depletion. The regulation of GlmS activity by EIIA^Ntr^ and the modulation of *glmS* translation by RapZ suggest that the genes comprising the *rpoN* operon play a key role in maintaining amino sugar homeostasis in response to nitrogen availability and the amino sugar concentration in the bacterial cytoplasm.

The phosphotransferase system (PTS) is a systematic bacterial device composed of a cascade of enzymes that transfer phosphate moieties derived from phosphoenolpyruvate (PEP) in sequential order. The canonical PTS that occurs in a wide range of bacteria is a sugar PTS called the phosphoenolpyruvate:carbohydrate PTS, which is comprised of enzyme I (EI), histidine phosphocarrier protein (HPr), and enzyme II complexes (EIIA, EIIB, EIIC, and sometimes EIID). The EIIA, EIIB, and EIIC proteins usually form substrate-specific transfer cascades with one membrane-spanning protein, EIIC in general, in contact with their extracellular substrates^1^. In contrast, EI and HPr are universal cytoplasmic proteins, which take-up diverse carbohydrates, such as sugars and their derivatives^1^. In addition to the uptake and concomitant phosphorylation of many carbohydrates, the PTS also conducts diverse regulatory functions, sensing available carbon sources^2^,^3^,^4^,^5^. The PTS accomplishes regulatory roles either by phosphorylating target proteins or by directly interacting with their target proteins. Proteins containing a specific PTS-recognizable phosphorylation domain are phosphorylated by the components of the PTS phosphorylation cascade and their activities are modulated. The phosphorylatable target proteins include a variety of non-PTS transporters and transcription regulators^5^. In the latter case, phosphorylated or unphosphorylated forms of the PTS proteins directly interact with target proteins, leading to activation or repression of their functions mainly involved in transport and transcription regulation^6^,^7^. EIIA^Glc^-mediated catabolite repression is a well-established regulatory mechanism by the PTS^8^,^9^. Unphosphorylated EIIA^Glc^ under glucose abundant conditions interacts with proteins necessary for the transport and metabolism of non-PTS carbohydrates, such as lactose, maltose, and glycerol, and inhibits their activities for PTS-catalyzed uptake of glucose as the preferred sugar. Interestingly, numerous *Proteobacteria* possess incomplete PTS cascades devoid of any known EIIB or EIIC proteins^5^. Thus, an incomplete PTS lacking substrate-specific EIIB and EIIC proteins is supposed to conduct regulatory functions by interacting with non-PTS substrates, instead of taking up sugar.

Extensive genome analysis revealed a paralog of the sugar PTS, called nitrogen PTS (PTS^Ntr^) in many *Proteobacteria*^10^. However, the repertoire is incomplete. In parallel with the sugar PTS, the PTS^Ntr^ possesses EI^Ntr^ (an EI paralog encoded by *ptsP*) and NPr (an HPr paralog encoded by *ptsO*), which catalyze phosphorylation of EIIA^Ntr^ (an EIIA^Mtl^ paralog encoded by *ptsN*) with a PEP-derived phosphoryl group but the PTS^Ntr^ is devoid of any known counterparts of the membrane-bound EIIB and EIIC enzymes, which transport extracellular substrates^10^,^11^,^12^. Accordingly, the PTS^Ntr^ has been speculated to conduct regulatory functions exclusively using EIIA^Ntr^ as the output regulator protein rather than a component of transport machinery^13^. An increasing number of data show that PTS^Ntr^ is associated with a plethora of cellular processes, including virulence^7^,^14^, nitrogen metabolism^10^,^15^, carbon metabolism^16^, K^+^ homeostasis^6^,^17^,^18^, and (p)ppGpp synthesis/hydrolysis^19^,^20^.

The *ptsN* gene encoding EIIA^Ntr^ is located downstream of *rpoN* in many *Proteobacteria*^21^. An alternative σ^54^ sigma factor encoded by *rpoN* participates in the expression of diverse genes and operons exclusively associated with nitrogen utilization and metabolism^22^. A genetic approach was employed on the *rpoN* operon containing *ptsN* to unravel the primary role of PTS^Ntr^ conserved in many bacteria during evolution and revealed that the ancestral *rpoN* operon composed of at least 11 genes has evolved to retain *rpoN*, *yhbH*, *ptsN*, *rapZ*, and *ptsO* in many *Gammaproteobacteria*^13^,^23^, suggesting a conserved function for the remaining genes. Localization of the *ptsN* and *ptsO* PTS^Ntr^ genes in the *rpoN* operon suggests a role for PTS^Ntr^ associated with nitrogen metabolism. Due to the absence of a component responsible for uptake and concomitant phosphorylation of a specific substrate, it has been unclear what stimuli determine the phosphorylation status of the PTS^Ntr^ components. However, it was recently revealed that EI^Ntr^ senses nitrogen availability through the glutamine (Gln) and α-ketoglutarate (α-KG) ratio and accordingly modulates the phosphorylation status of the EIIA^Ntr^ output regulator in *E. coli*^24^. Nitrogen is prerequisite for producing proteins, nucleic acids, and cell wall constituents. Therefore, it is critical for bacteria to sense availability of cellular nitrogen sources and allot them depending on the circumstances. The functional relevance of the genes comprising the *rpoN* operon to nitrogen utilization for cell wall construction has been frequently demonstrated. NPr encoded by *ptsO* decreases biosynthesis of lipid A in the lipopolysaccharides (LPS) layer by inhibiting LpxD activity and blocking the inflow of UDP-GlcNAc into cell wall constituents^25^. RapZ encoded by *rapZ* negatively controls synthesis of the D-glucosamine-6-phosphate synthase (GlmS) by triggering decay of GlmZ, a small RNA facilitating *glmS* translation^26^. GlmS is a key enzyme in the LPS and peptidoglycan biosynthetic pathways. The association between σ^54^, a transcription factor encoded by *rpoN*, and bacterial exterior constitution has been observed in many bacteria^27^,^28^. Co-clustering of PTS^Ntr^ genes with *rpoN* and convergent negative roles of the *rpoN* operon genes during synthesis of cell envelope constituents have led to exploring a concordant role for EIIA^Ntr^ in cell envelope formation in response to nitrogen abundance. In this study, we discovered direct protein-protein interaction between EIIA^Ntr^ and GlmS and suggest a novel role for *ptsN* regulating amino sugar biosynthesis, which is contextually associated with adjacent genes.

## Results

### Specific interaction between Enzyme IIA^Ntr^ and glucosamine-6-phosphate synthase (GlmS) in *Salmonella* Typhimurium

**Figure 1:**
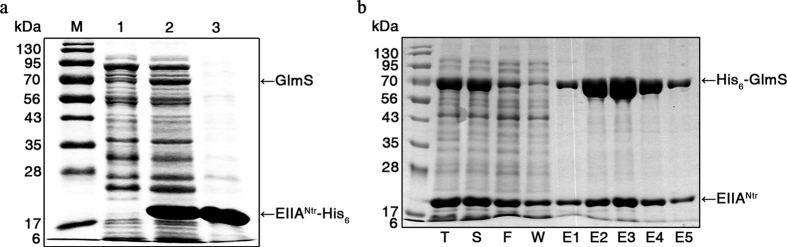
EIIA^Ntr^ specifically binds to glucosamine-6-phosphate synthase (GlmS). (**a**) Ligand-fishing using EIIA^Ntr^-His_6_ as a bait. Total cell extract from *S*. Typhimurium SL1344 culture was incubated with EIIA^Ntr^-His_6_ or not. The proteins eluted from Ni-NTA resin (lane 1, whole cell extract only; lane 2, whole cell extract mixed with EIIA^Ntr^-His_6_) and purified EIIA^Ntr^-His_6_ (lane 3) were analyzed by SDS-PAGE. The protein band indicated by the arrow in lane 2 was identified as GlmS by LC-MS/MS analysis. Size markers (M) in kDa are aligned at left. (**b**) Co-purification of EIIA^Ntr^ and GlmS *in vivo*. *E. coli* BL21 (DE3) harboring pETDuet-*glmS*·*ptsN* was geneticallymanipulated to produce EIIA^Ntr^ and His_6_-tagged GlmS simultaneously by isopropyl β-D-1-thiogalactoside (IPTG) addition. EIIA^Ntr^ bound to His_6_-GlmS was co-purified using Ni-NTA metal-affinity resin. Proteins analyzed in SDS-PAGE are aliquots from total cell extracts (T), supernatant after centrifuging the cell extracts (S), column flow-through (F), wash (W), and the five elution fractions (E1–E5). Molecular masses of standards are presented in kDa on the left.

Although the general sugar PTS components exert regulatory functions by either phosphorylating or interacting with target proteins, PTS^Ntr^ EIIA^Ntr^ seems to have a bias toward interaction-mediated regulation. Many studies have demonstrated direct interactions between unphosphorylated EIIA^Ntr^ and diverse proteins involved in K^+^ transport^6^,^17^, virulence regulation^7^, and the ppGpp-mediated stringent response^19^,^20^. A common theme of these EIIA^Ntr^ interactions is that unphosphorylated EIIA^Ntr^ prevails during the protein-protein interaction. To search for target proteins interacting with EIIA^Ntr^, EIIA^Ntr^ tagged with His_6_ at its C-terminus (EIIA^Ntr^-His_6_) was isolated in its unphosphorylated form and used as bait in a ligand-fishing strategy with *Escherichia coli* as a representative *Gammaproteobacteria*^6^,^21^. Here, to archive more diversity in binding partners, *Salmonella enterica* serovar Typhimurium, a member of *Enterobacteriaceae* with high genetic similarity to *E. coli*, was chosen. EIIA^Ntr^-His_6_ was incubated with a crude protein extract of *Salmonella* Typhimurium SL1344, and the proteins bound to EIIA^Ntr^-His_6_ were pulled down with a metal-affinity resin. The sodium dodecyl sulfate-polyacrylamide gel electrophoresis (SDS-PAGE) analysis of isolated protein complexes from ligand-fishing experiments revealed a protein band of approximately 70 kDa that specifically co-precipitated with EIIA^Ntr^-His_6_ (Fig. 1a). Liquid chromatography-tandem mass spectrometry (LC-MS/MS) peptide mapping identified this protein as GlmS. Other proteins predicted together with GlmS in the LC-MS/MS analysis are listed in Supplementary Table S2. The specific interaction between EIIA^Ntr^ and GlmS was further validated *in vivo.* His_6_-GlmS and EIIA^Ntr^ were produced simultaneously under the *lac* promoter of the pETDuet plasmid in *E. coli*, and the crude protein extract was passed through a Ni-NTA resin column. EIIA^Ntr^ was pulled down together with His_6_-GlmS (Fig. 1b), indicating direct interaction between EIIA^Ntr^ and GlmS *in vitro* and *in vivo*.

### Phosphorylation status of EIIA^Ntr^ influences its binding affinity with GlmS

An intimate association between PTS^Ntr^ and nitrogen metabolism has been proposed undoubtedly through genetic localization of *ptsN* and *ptsO* in the *rpoN* operon and multiple experimental results. The intracellular Gln: α-KG ratio indicates the balance between nitrogen and carbon sources and controls the regulatory circuit assigned to assimilate extracellular ammonia into Gln^29^ and the phosphorylation status of PTS^Ntr^. The EI^Ntr^ GAF domain senses the abundance of nitrogen through a high Gln/α-KG ratio, dampens autophosphorylation of EI^Ntr^, and dephosphorylates EIIA^Ntr^ in turn^24^. Other *ptsO* mutants unable to phosphorylate EIIA^Ntr^ reduce the expression of genes required for nitrogen assimilation and glutamine synthetase, suggesting a negative role for unphosphorylated EIIA^Ntr^ during nitrogen incorporation into Gln^30^,^31^. It is plausible that EIIA^Ntr^ responding to nitrogen availability orchestrates the rate of nitrogen acquisition and its utilization in other cellular components. However, it remains unresolved which molecule interacts with phosphorylated or unphosphorylated EIIA^Ntr^ in the nitrogen metabolic and utilization pathways. The finding of an interaction between EIIA^Ntr^ and GlmS may provide a clue to define the role of PTS^Ntr^ in nitrogen metabolism, as GlmS (D-glucosamine-6-phosphate synthase, EC 2.6.1.16) exploits Gln to produce equimolar D-glucosamine-6-phosphate (GlcN6P), lowering intracellular nitrogen resources.

**Figure 2:**
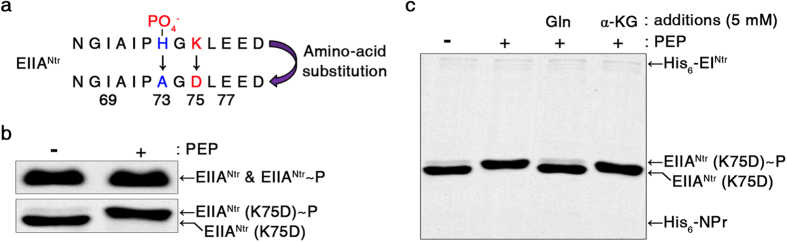
EIIA^Ntr^ phosphorylation status is influenced by nitrogen abundance. (**a**) EIIA^Ntr^ modification strategy. The number of SDS molecules bound to a protein is affected by the charge of the amino acids around the phosphorylation site, which can change the mobility of the protein on SDS-PAGE^24^,^54^. A histidine (73) amino acid of EIIA^Ntr^ was changed to alanine (73) to construct the unphosphorylated form of EIIA^Ntr^ (H73A), and a lysine (75) was substituted for aspartic acid (75) to provide negative charge effects on EIIA^Ntr^. (**b**) Phosphorylation-dependent upshift of EIIA^Ntr^ (K75D). The intact form of EIIA^Ntr^ and its EIIA^Ntr^ (K75D) mutant derivative were incubated with or without 1 mM PEP under phosphorylating conditions and then analyzed by SDS-PAGE. EIIA^Ntr^ (K75D) showed excellent phosphorylation-dependent mobility shift (PDMS), whereas the intact form of EIIA^Ntr^ showed comparable mobility independent of its phosphorylation. (**c**) Differential phosphorylation status of EIIA^Ntr^ between metabolites. The phosphorylation-dependent mobility shift of EIIA^Ntr^-His_6_ (K75D) was assessed using different metabolites. EIIA^Ntr^-His_6_ (K75D) was incubated with PEP, His_6_-EI^Ntr^, and His_6_-NPr in the presence of glutamine (Gln) or α-ketoglutarate (α-KG). The phosphorylation levels of EIIA^Ntr^-His_6_ (K75D) were compared by SDS-PAGE.

**Figure 3:**
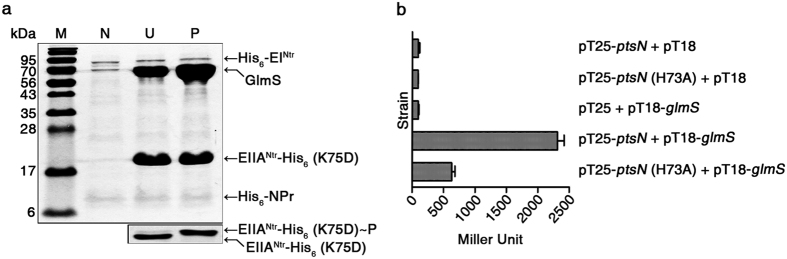
Phosphorylation status of EIIA^Ntr^ influences the binding affinity between EIIA^Ntr^ and GlmS. (**a**) Increased binding affinities of EIIA^Ntr^-His_6_ (K75D) to GlmS after phosphorylation. Phosphorylated and unphosphorylated forms of EIIA^Ntr^-His_6_ (K75D) were incubated with equivalent amounts of GlmS, and the levels of GlmS bound to phosphorylated (P) or unphosphorylated (U) EIIA^Ntr^-His_6_ (K75D) were compared (top). PEP-dependent phosphorylation of EIIA^Ntr^-His_6_ (K75D) was verified in parallel (bottom inlet). Line (N) does not contain either the EIIA^Ntr^-His_6_ (K75D) or the GlmS protein as a control. (**b**) Differential interaction between EIIA^Ntr^ and GlmS depending on EIIA^Ntr^ phosphorylation status *in vivo*. Protein-protein interactions between EIIA^Ntr^ and GlmS were verified using a bacterial two-hybrid system. Plasmid pKT25 containing *ptsN* or *ptsN* (H73A) and plasmid pUT18 harboring *glmS* were introduced into a reporter strain respectively or in combination. The reporter strains were cultivated in LB broth supplemented with IPTG, and β-galactosidase activity was determined to examine the strength of the protein-protein interactions. This experiment was performed in triplicate.

As demonstrated in other EIIA^Ntr^ binding partners, we assumed that the unphosphorylated status of EIIA^Ntr^ was preferred during complex formation with GlmS. Unphosphorylated EIIA^Ntr^ under high Gln concentrations was likely to bind GlmS and stimulate its GlcN6P production with Gln consumption. However, to our surprise, the binding affinity of EIIA^Ntr^ to GlmS was enhanced by phosphorylation. For this observation, *Salmonella* EIIA^Ntr^-His_6_ was modified to EIIA^Ntr^-His_6_ (K75D) (Fig. 2a) to exhibit a phosphorylation-dependent mobility shift (PDMS) on SDS-PAGE without changing its functional properties (Supplementary Fig. S1), as established in *E. coli*^24^. *Salmonella* EIIA^Ntr^-His_6_ (K75D) showed an upshift in mobility after PEP-derived phosphorylation (Fig. 2b) and was downshifted by adding Gln, indicating its unphosphorylated state under excessive nitrogen concentrations (Fig. 2c), as expected. When phosphorylated or unphosphorylated EIIA^Ntr^-His_6_ (K75D) was incubated with GlmS, phosphorylated EIIA^Ntr^ recruited more GlmS than did the unphosphorylated form in a concentration-dependent manner but EIIA^Glc^ used as a control did not interact (Fig. 3a and Supplementary Fig. S2A). The possibility of non-specific interaction of GlmS with anti-His_6_ antibody or Ni-NTA resin was ruled out as detailed in Supplementary Fig. S3A,B.

The differential binding affinity of EIIA^Ntr^ to GlmS due to its phosphorylation status was further verified *in vivo* using a bacterial two-hybrid system^32^. EIIA^Ntr^ and GlmS fused to two different sub-domains of *Bordetella pertussis* adenylate cyclase bound each other and elevated cAMP levels. In accordance with the *in vitro* observations, EIIA^Ntr^ (H73A), a derivative with a mutation at the phosphorylation site^33^, caused lower cAMP levels than that of intact EIIA^Ntr^ (Figs 2a and 3b). This result was also confirmed by the different color intensities between bacterial cultures producing phosphorylatable or unphosphorylatable EIIA^Ntr^ in the medium containing 5-bromo-4-chloro-3-indolyl-β-D-galactoside (X-gal) (Supplementary Fig. S2B).

### EIIA^Ntr^ inhibits GlmS activity

**Figure 4:**
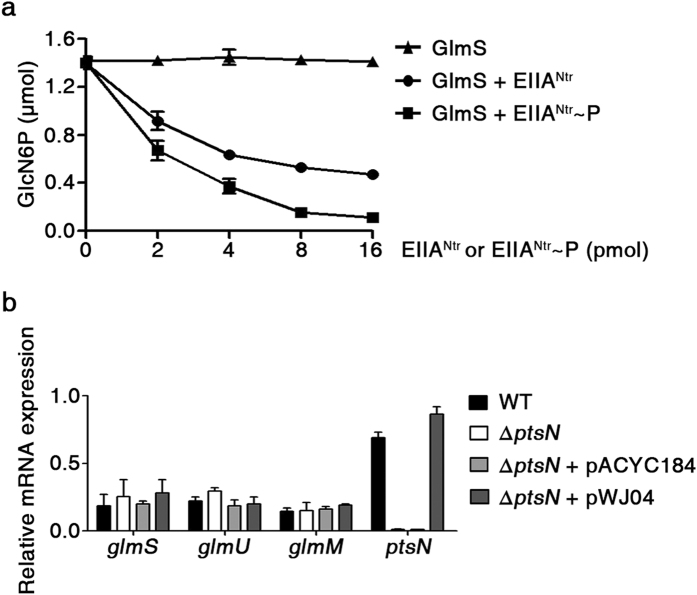
EIIA^Ntr^ inhibits GlmS-mediated GlcN6P production. (**a**) Negative effects of EIIA^Ntr^ on GlcN6P production. GlmS was incubated with different amounts of phosphorylated or unphosphorylated EIIA^Ntr^-His_6_ (K75D) (0–16 pmol) and GlcN6P production was measured by HPLC. The results from triplicates are plotted. (**b**) No effect of EIIA^Ntr^ on the expression of genes involved in amino sugar metabolism. Wild-type and Δ*ptsN* mutant strains were transformed with pWJ04 containing *ptsN* and its presumable promoter, and qRT-PCR was conducted to compare mRNA levels of *glmS*, *glmU, glmM*, and *ptsN*. All mRNA levels were normalized using *gyrB*, and the relative expression ratios were averaged from three independent measurements.

EIIA^Ntr^, unlike other interactions with TrkA^6^, KdpD^17^, E1 of pyruvate dehydrogenase^33^, and SsrB^7^, prefers the phosphorylation state to interact with GlmS. To decipher the link between PTS^Ntr^ and nitrogen or amino acid metabolism, the influence of EIIA^Ntr^ on GlmS activity was studied. EIIA^Ntr^ is known to exert both positive and negative effects on the roles of partner proteins^17^,^33^. GlmS converts D-fructose-6-phosphate (Fru6P) into GlcN6P by hydrolyzing Gln to glutamate (Glu). An enzymatic assay for GlmS was set up using Gln, Fru6P, and GlmS, and GlcN6P production was measured by high performance liquid chromatography (HPLC) (Supplementary Fig. S4A,B). Then, GlmS was incubated with the phospho- or unphospho-forms of EIIA^Ntr^-His_6_ (K75D) and GlcN6P production was compared (Fig. 4a). Adding EIIA^Ntr^-His_6_ (K75D) decreased GlcN6P production, and its phosphorylation caused a more drastic reduction of GlcN6P production, indicating a negative role of EIIA^Ntr^ in GlmS activity.

GlmS exerts negative feedback regulation in response to GlcN6P. Excessive GlcN6P production stimulates degradation of *glmS* mRNA^26^,^34^. To define the negative effect of EIIA^Ntr^ on amino sugar production in detail, the levels of mRNAs relevant to amino sugar metabolism were evaluated in the presence or absence of EIIA^Ntr^. Quantitative reveres transcription-polymerase chain reaction (qRT-PCR) revealed that the mRNA levels of *glmS*, *glmM*, and *glmU* required to convert Fru6P to uridinediphospho-*N*-acetylglucosamine (UDP-GlcNAc), a main amino sugar substrate for cell wall structure, were not affected by EIIA^Ntr^ (Fig. 4b), indicating that EIIA^Ntr^ modulates GlmS activity by protein-protein interaction and not by transcriptional regulation.

### EIIA^Ntr^ affects the GlmS-mediated bacterial growth rate

**Figure 5:**
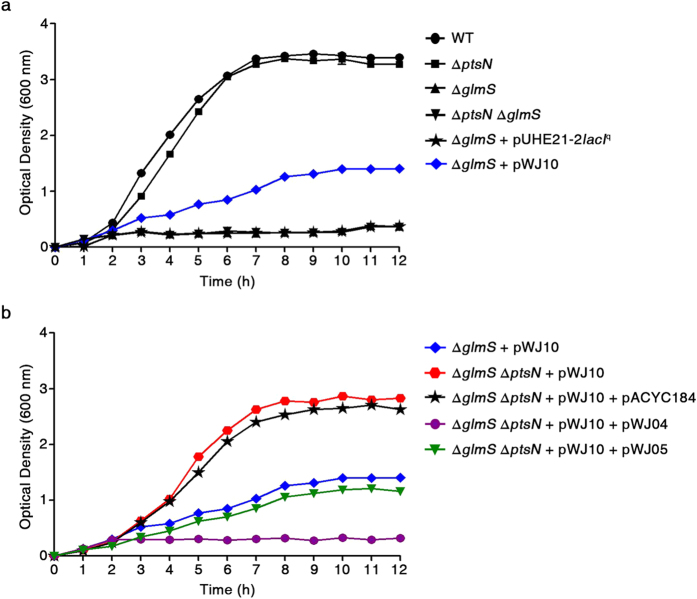
*Salmonella* growth is influenced by the interaction between EIIA^Ntr^ and GlmS. (**a**) The absence of *glmS* is lethal to bacteria. Effects of GlmS and EIIA^Ntr^ on bacterial growth were assessed individually or in combination. Introduction of pWJ10 (a pUHE21-2*lacI*^q^::*glmS*) providing GlmS in *trans* partially restored the growth defect in Δ*glmS* mutant. GlmS production level was titrated using 10 μM IPTG (Supplementary Fig. S5). (**b**) Modulation of growth rate by the interaction between EIIA^Ntr^ and GlmS. Using the Δ*glmS* mutant complemented with pWJ10 as a parent strain, the effect of the EIIA^Ntr^ interaction with GlmS on bacterial growth was evaluated by deleting the chromosomal *ptsN* gene and introducing pWJ04 and pWJ05, which provided EIIA^Ntr^ and EIIA^Ntr^ (H73A), respectively. All growth measurements in (**a,b**) were performed in triplicate, and the average optical densities at 600 nm are plotted.

Inhibiting GlmS activity through the interaction with EIIA^Ntr^ was further confirmed *in vivo.* GlmS is a key enzyme in the production of amino sugars, which are essential precursors for bacterial cell wall peptidoglycans and LPS in the outer membrane of Gram-negative bacteria. Therefore, bacteria deprived of functional GlmS suffer from attenuated growth due to a lack of building blocks for constructing the cell wall when exogenous amino sugars are depleted^35^,^36^. To examine whether growth of *Salmonella* is attenuated by the interaction between EIIA^Ntr^ and GlmS, a balanced-lethal system was established in which the absence of chromosomal *glmS* was partially complemented by controlled expression of *glmS* on the pWJ10 plasmid containing the *lac* promoter (Supplementary Fig. S5). A Δ*glmS* mutant strain with a severe growth defect due to the lack of cell envelope components^37^ was supplemented with pWJ10 in the presence of 10 μM IPTG, showing approximately 50% growth of wild-type *Salmonella* (Fig. 5a). The additional deletion of the *ptsN* gene in the Δ*glmS* mutant containing pWJ10 restored growth to a level comparable to that of wild-type cells, suggesting that GlmS was relieved of the inhibition by EIIA^Ntr^. Accordingly, introducing plasmids producing intact EIIA^Ntr^ or its derivative, EIIA^Ntr^ (H73A), re-repressed the growth of the Δ*glmS* Δ*ptsN* strain containing pWJ10. As expected, EIIA^Ntr^ (H73A), which is incapable of phosphorylation in response to stimuli^15^, showed a less inhibitory effect than the intact form probably due to diminished binding affinity to GlmS (Fig. 5b). Examination of viable cell number under the same condition showed the consistent result with that of bacterial growth rate analysis described above (Supplementary Fig. S6).

These results verify that EIIA^Ntr^-mediated GlmS inhibition might lead to a growth defect attributable to the impaired integrity of the cell wall and further imply a role for EIIA^Ntr^ controlling the production rate of amino sugars depending on nitrogen accessibility.

### EIIA^Ntr^ and GlmS interact to form a heterotrimeric complex

**Figure 6:**
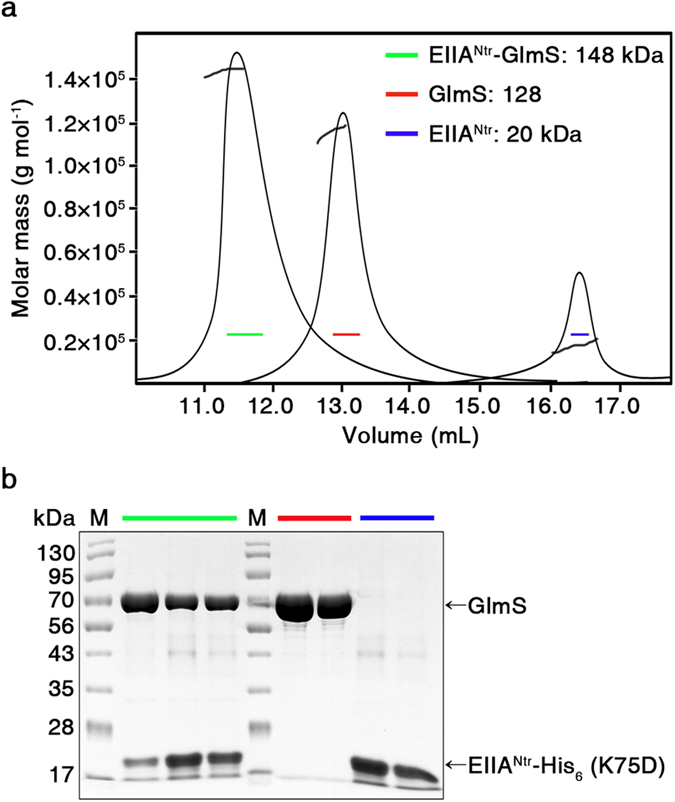
Trimeric complex is formed between EIIA^Ntr^ and GlmS. (**a**) Stoichiometric analysis of the EIIA^Ntr^ and GlmS complex. A solution of the EIIA^Ntr^ and GlmS complex was analyzed by SEC-MALS. The molecular mass of GlmS alone in solution was 128 kDa, indicating the active dimer, and that of EIIA^Ntr^ was 20 kDa, indicating the monomeric form. A macromolecule with a peak of 148 kDa appeared in the solution composed of EIIA^Ntr^ and GlmS, and the size was accordant with a EIIA^Ntr^-GlmS_2_ heterotrimer composed of two GlmS molecules (128 kDa) and one EIIA^Ntr^ (20 kDa). (**b**) Identifying the eluted fractions by SDS-PAGE. The SEC-MALS eluted fractions from the 148, 128, and 20 kDa proteins, which were presumably the EIIA^Ntr^-GlmS_2_ complex, GlmS dimer, and the EIIA^Ntr^ monomer, respectively, were analyzed by SDS-PAGE and Coomassie Blue staining. Green, red, and blue lines indicate the 148, 128, and 20 kDa fractions, respectively.

A cascade of enzymes, including GlmS, GlmM, and GlmU are required for *bona fide* amino sugar synthesis. GlmS, which is responsible for the first and rate-limiting step in the hexosamine pathway, converts Fru6P into GlcN6P. GlcN6P is subsequently modified to glucosamine-1-phosphate (GlcN1P) by GlmM and further converted to UDP-GlcNAc by GlmU^38^,^39^,^40^. However, in the presence of abundant exogenous *N*-acetylglucosamine (GlcNAc) transported via NagE and ManXYZ, the GlmS-catalyzed reaction is bypassed, whereas GlmM and GlmU are consistently required for its conversion into UDP-GlcNAc^41^. Hence, the activity of GlmS is selectively shut down in response to amino sugar availability. As *glmS* and *glmU* constitute the *glmUS* operon and their transcripts are strictly coupled^42^, there should be a regulatory mechanism differentiating synthesis and activity of GlmS from those of GlmU after transcription. For example, translation of *glmS* mRNA is separately controlled by the GlmY/GlmZ/RapZ system, which responds to the cellular concentration of GlcN6P^26^, as described in detail below. In addition, GlmS changes its structure from an active dimer to an inactive hexamer as its cellular concentration increases and GlcN6P is accumulated as the product^43^. To refine the interaction between EIIA^Ntr^ and GlmS in greater detail, we determined the stoichiometric mass-action model in the EIIA^Ntr^ and GlmS complex. We first assumed the architectural contribution of EIIA^Ntr^ toward converting the GlmS structure into the inactive hexameric form: EIIA^Ntr^ binding likely helps GlmS molecules conglomerate in an inactive form without repulsion or stabilizes the inactive polymeric structure to prevent its return to the active dimer form. To examine this possibility, EIIA^Ntr^ and GlmS in solution were subjected to size exclusion chromatography with multi-angle light scattering (SEC-MALS) in combination or individually (Fig. 6a). GlmS peaked at about 128 kDa, which is equivalent to the mass of the dimeric form of GlmS, and EIIA^Ntr^ was maintained as a monomer, showing a peak of around 20 kDa in solution. During SEC, the MALS detected a large molecule of approximately 148 kDa in the solution containing GlmS and EIIA^Ntr^, suggesting two molecules of GlmS obstructed by a molecule of EIIA^Ntr^. The peaks were further dissected by SDS-PAGE to define the protein composition (Fig. 6b). These results suggest that EIIA^Ntr^ inhibits GlmS by binding to its active dimeric form but is less likely to contribute to conversion of GlmS into the inactive hexameric form.

### EIIA^Ntr^ stability is modulated by Lon in response to amino sugar availability

**Figure 7:**
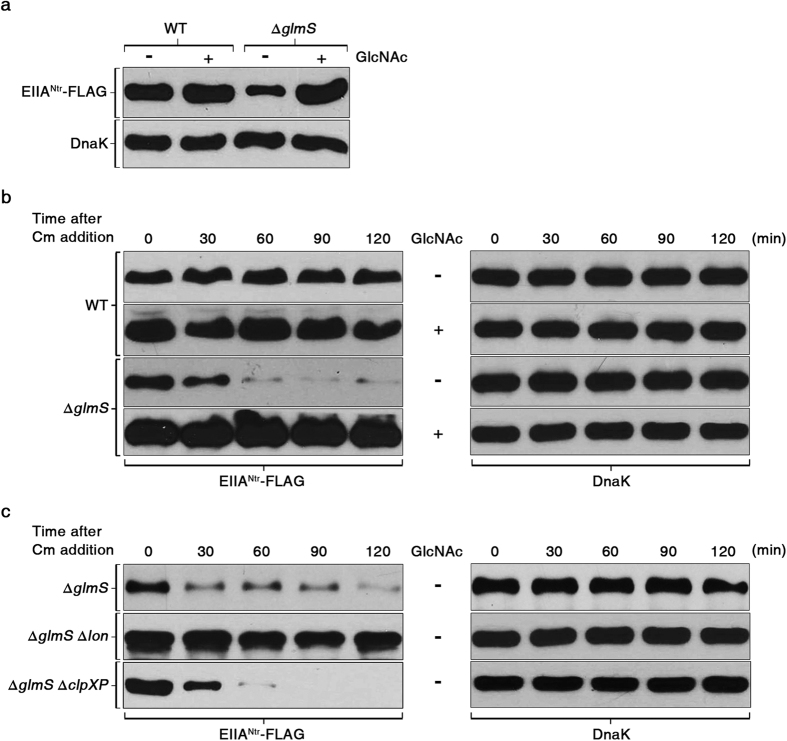
EIIA^Ntr^ degradation is controlled by Lon protease in response to amino sugar availability. (**a**) Comparison of EIIA^Ntr^-FLAG protein levels between wild-type and Δ*glmS* mutant strains. Protein samples were isolated from wild-type and Δ*glmS* mutant strains in the presence or absence of GlcNAc 3 h post inoculation as described in Supplementary Figs S8A and S10, and subjected to Western blot analysis to compare the EIIA^Ntr^-FLAG levels between conditions. (**b**) Effect of amino sugar availability on stability of EIIA^Ntr^-FLAG. Chloramphenicol was added to the cultures of the wild-type and Δ*glmS* mutant strains at 3 h as described above, and total proteins harvested every 30 min were used to assess the EIIA^Ntr^ and DnaK decay rates. (**c**) Comparison of the stability of EIIA^Ntr^-FLAG in the absence of Lon or ClpXP protease in the Δ*glmS* mutant strain. The Δ*glmS*, Δ*glmS* Δ*lon*, and Δ*glmS* Δ*clpXP Salmonella* strains were cultured under conditions identical to those used above, and the level of EIIA^Ntr^-FLAG and DnaK was assessed in each strain using Western blotting every 30 min after adding chloramphenicol. In all experiments, equivalent amounts of total protein were loaded in each lane for SDS-PAGE and DnaK levels were measured in parallel to verify equivalent total protein amounts between lanes.

Maintaining GlcN6P homeostasis has been ascribed to the GlmY/GlmZ/RapZ system^24^. RapZ inhibits GlmS expression by accelerating the decay of the small RNA GlmZ, which facilitates *glmS* translation. However, bacteria sensing an amino sugar deficiency exploit GlmY to antagonize GlmZ degradation by RapZ. Co-localization of *ptsN* and *rapZ* in the *rpoN* operon reinforces the concordant role of these two genes in GlmS-mediated regulation of cell envelope integrity. We suspected that amino sugar was the signal triggering coordinated negative regulation of EIIA^Ntr^ and RapZ on GlmS. However, *ptsN* and *rapZ* mRNA levels were not affected by amino sugar availability, whereas *glmS* transcription was negatively feedback regulated by ample amino sugars, as reported previously^26^,^44^ (Supplementary Figs S7A,B and S8A,B). Instead, intriguingly, the level of the EIIA^Ntr^ protein decreased when GlcN6P production stopped due to the lack of GlmS and when no exogenous GlcNAc, a metabolite substitute for GlcN6P^26^,^44^,^45^ was supplied (Fig. 7a). In contrast, adding GlcNAc increased the levels of EIIA^Ntr^ regardless of the presence of GlmS (Fig. 7a). These results indicate that intracellular amino sugar availability controls EIIA^Ntr^ at the protein level.

To further explore modulation of intracellular EIIA^Ntr^ concentration depending on amino sugar availability, the rate of EIIA^Ntr^ proteolysis was compared between amino sugar-abundant and -depleted conditions. To prevent *de novo* protein synthesis, chloramphenicol was added to the culture 3 h post-inoculation when amino sugar availability influenced bacterial growth rate (Supplementary Fig. S9)^26^,^46^. The Δ*glmS* mutant strain depleted of amino sugars rapidly degraded EIIA^Ntr^-FLAG but retained EIIA^Ntr^-FLAG at high levels for at least 2 h when provided with excessive GlcNAc, indicating regulation of the EIIA^Ntr^ proteolysis rate by amino sugar availability (Fig. 7b). Lon and Clp proteases in *E. coli* and other bacteria are responsible for 70–80% of energy-dependent protein degradation^47^,^48^. We observed that deleting *lon* quenched the programmed proteolysis of EIIA^Ntr^-FLAG upon amino sugar depletion in the Δ*glmS* mutant strain (Fig. 7c). In this experiment, DnaK levels were measured in parallel to verify equivalent total protein amounts between lanes, and a similar test using RpoB, which was used as an additional control instead of DnaK, was also conducted (Supplementary Fig. S10). These results indicate that Lon accelerates EIIA^Ntr^ degradation when *Salmonella* lacks amino sugar metabolites.

## Discussion

Nitrogen is essential to every living organism, including bacteria; it is assimilated into amino acids and further into proteins, which constitute cellular components that conduct diverse biological activities. Nitrogen is also used in amino sugar compounds, the structural residues of bacterial cell walls. The nitrogen moiety of Gln is integrated into Fru6P by GlmS, producing GlcN6P. GlcN6P is further converted to UDP-GlcNAc by a cascade of cytoplasmic enzymes, such as GlmM and GlmU. UDP-GlcNAc is an essential structural building block for peptidoglycans and LPS in bacterial cell walls. Therefore, the balance between synthesis and decomposition of amino sugars is directly influenced by the availability of carbon and nitrogen.

**Figure 8:**
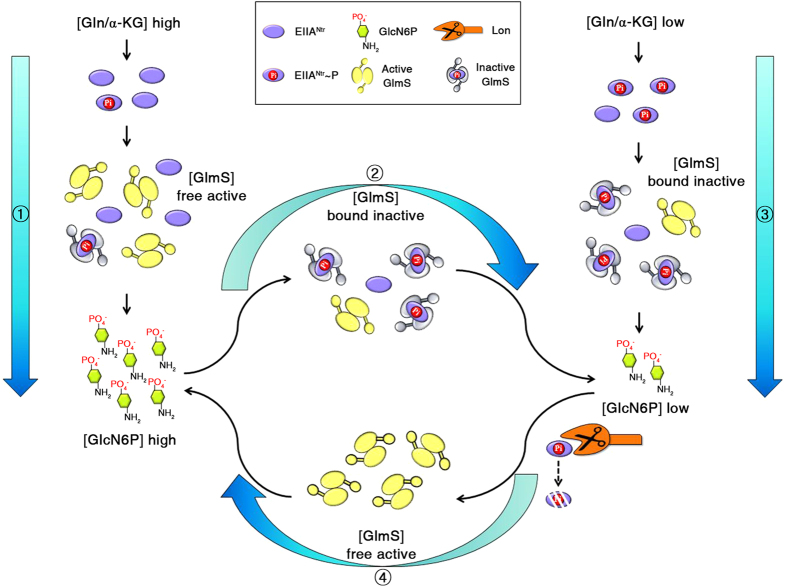
Proposed regulatory model for the control of GlmS activity by EIIA^Ntr^ in response to nitrogen and amino sugar availability. At high cellular glutamine (Gln) concentrations, EIIA^Ntr^ tends to be unphosphorylated and liberates the active form of GlmS that supplies GlcN6P and accelerates synthesis of the bacterial cell envelope (①). When GlcN6P is present in excess (②) or when Gln availability is restricted (③), phosphorylated EIIA^Ntr^ binds GlmS with increased affinity and inhibits its activity. The depletion of pre-existing GlcN6P leads to EIIA^Ntr^ degradation by Lon protease, freeing the active form of GlmS to supplement the lack of GlcN6P and maintain amino sugar homeostasis (④).

Cells under excessive nitrogen convert α-KG to Glu and further to Gln^49^. Gln is utilized as a nitrogen source under nitrogen-limiting conditions^50^. Hence, the Gln: α-KG ratio is generally used to predict the cellular balance between nitrogen and carbon abundance: high Gln/α-KG values under nitrogen-rich conditions vs. low Gln/α-KG values under nitrogen-depleted conditions. Bacteria must promptly recognize detrimental changes, such as nutrient deprivation, and adjust metabolic processes for their adaptation. For example, it has been recently revealed that *Caulobacter crescentus* triggers the phosphorylation of EI^Ntr^, the first enzyme of PTS^Ntr^ in response to low glutamine concentrations and subsequently phosphorylates EIIA^Ntr^, which in turn inhibits the hydrolase activity of SpoT by directly binding to SpoT. Modulation of SpoT activity by PTS^Ntr^ influences the cellular accumulation of (p)ppGpp, an alarmone controlling bacterial cell cycle progression and growth^20^. A similar role of PTS^Ntr^ sensing the nitrogen availability and modulating cellular metabolism was disclosed in this study. GlmS is the rate-limiting enzyme that consumes a molecule of Gln to synthesize an equivalent amount of the amino sugar GlcN6P. Thus, GlmS activity is tightly controlled in response to the availability of cellular nitrogen and amino sugars. Based on our results, we propose a regulatory circuit wherein EIIA^Ntr^ fine-tunes GlmS activity by assessing the abundance of nitrogen and amino sugars (Fig. 8). In the presence of sufficient nitrogen, dephosphorylated EIIA^Ntr^ does not significantly compromise GlmS activity, which, in turn, provides abundant amino sugars for constructing cell walls. When cells suffer from depleted nitrogen, phosphorylated EIIA^Ntr^ tightly binds to GlmS and inhibits the enzyme from consuming Gln to produce GlcN6P, which slows down LPS and peptidoglycan synthesis. Not only nitrogen availability but also amino sugar abundance modulates the influence of EIIA^Ntr^ on GlmS activity. Bacteria supplemented with abundant amino sugars, such as GlcNAc, delay the decay of EIIA^Ntr^ and subsequently decelerate *bona fide* amino sugar synthesis via GlmS. However, when the supply of exogenous amino sugars is suspended and the GlcN6P remnants are exhausted, Lon accelerates EIIA^Ntr^ proteolysis and relieves GlmS to supplement the lack of cellular amino sugars.

GlmS is a pivotal enzyme for maintaining cell wall integrity and employs multiple latch devices for regulation. GlmS maintains its own structural equilibrium between the active dimeric conformation and the inactive hexameric state by shifting toward the inactive hexameric form when its concentration increases and the GlcN6P product accumulates^43^. In addition to the conformational dynamics of GlmS, its translation is also controlled by the coordinated action between two regulatory GlmY and GlmZ small RNAs and the RapZ RNase adaptor protein. Following dissociation of the *glmS* monocistronic transcript from the *glmUS* cotranscript by RNaseE-mediated processing, GlmZ base-pairs with *glmS* mRNA and the aid of Hfq activates translation of *glmS* mRNA^51^. GlmZ turnover is determined by binding with RapZ, which recruits RNase E to the complex to facilitate GlmZ degradation. However, under low cellular GlcN6P concentrations, GlmY, with a secondary structure similar to GlmZ, increases and sequesters RapZ from GlmZ as a decoy, liberating GlmZ to activate *glmS* translation and subsequent GlcN6P production^26^. The results in our study established that GlmS is not only controlled at the post-transcriptional level by GlmY/GlmZ/RapZ but is also coordinated at the post-translational level by EIIA^Ntr^ and Lon. PTS^Ntr^ senses accessibility of nitrogen and controls the phosphorylation status of EIIA^Ntr^. Phosphorylated or unphosphorylated EIIA^Ntr^ hampers catalytic GlmS activity by binding to the active duplex form with different affinities. However, GlmS is released from the inhibition by Lon-mediated degradation of EIIA^Ntr^ upon depletion of cellular amino sugars.

Because of the incomplete architecture of PTS^Ntr^ lacking the phosphate recipient coupled with EIIA^Ntr^, EIIA^Ntr^ has been speculated to serve as a substantive regulator, and its pleiotropic regulation has been observed in diverse cellular processes, including nitrogen metabolism, K^+^ homeostasis, and interactions with host cells^7^,^17^,^33^. However, the pleiotropic regulatory effects of PTS^Ntr^ may not be completely attributable to the direct interactions between PTS^Ntr^ components and cellular targets; rather, many of the regulatory outputs might be secondarily caused by a few unmediated processes operated by EIIA^Ntr^. K^+^ homeostasis controlled by direct binding of EIIA^Ntr^ to TrkA and KdpD would explain such a mechanistic link^6^,^17^. The cellular K^+^ concentration managed by EIIA^Ntr^ could be a manifold signal leading to activation or inhibition of enzymes, transcriptional regulation, and pH homeostasis^13^.

Similarly, additional direct targets of EIIA^Ntr^ may yet to be identified, and GlmS is one of them. Direct regulation of GlmS by EIIA^Ntr^ discloses a sophisticated regulatory circuit balancing metabolite fluxes among carbon, nitrogen, and amino sugars. The possibility of a metabolic link between carbon and nitrogen has been undoubtedly raised. The sugar PTS and nitrogen PTS cross-talk by phosphorylating counterpart components^10^,^52^. α-KG has been identified as a key molecule balancing carbon and nitrogen assimilation and controlling EIIA^Ntr^ regulatory activity. A challenge for bacteria living in fluctuating nutritional conditions is to notice the accessibility of essential nutrients, such as carbon and nitrogen, and determine the flux rates from nutrients to energy production and building biomass. Doucette *et al*. observed that a sudden increase in nitrogen availability led to an immediate glucose uptake and discovered that α-KG, which accumulates under nitrogen-depleted conditions, inhibits the autophosphorylation of enzyme I (EI) of sugar PTS and blocks the entry of glucose, synchronizing carbon uptake with nitrogen availability^49^. α-KG which is accumulated in nitrogen-limiting conditions, in turn, binds to the GAF domain of NifA, a transcriptional activator for nitrogen fixation (*nif*) genes, and triggers nitrogen uptake into the cell^53^. Cells fortified with nitrogen convert α-KG to Glu and further to Gln and subsequently resume glucose uptake by releasing the sugar PTS EI from α-KG inhibition. Intriguingly, the GAF domain responding to α-KG is also possessed by EI^Ntr^ of PTS^Ntr^ and a nitrogen deficiency is directly delivered to EIIA^Ntr ^54, which dampens GlmS activity, thereby shutting down inflow of nitrogen into amino sugars. Thus, one could speculate that α-KG serves as an interface signal integrating cellular physiological status and coordinating carbon and nitrogen assimilation; physiological status is further transformed to EIIA^Ntr^ across the PTS^Ntr^ phosphorelay system, which accommodates amino sugar biosynthesis on demand.

It was observed that the unphosphorylated form of EIIA^Ntr^ is exclusively dominant during rapid growth of *Pseudomonas putida*, a member of *Gammaproteobacteria*, and suggested that EIIA^Ntr^ phosphorylation status might be influenced by cellular physiological status representing the availability of carbon and nitrogen^55^. However, it is unknown how phosphorylation of EIIA^Ntr^ alters bacterial fitness during environmental adaptation. Our results suggest that EIIA^Ntr^, which is phosphorylated under nitrogen-limiting conditions, compromises *bona fide* amino sugar biosynthesis by inhibiting GlmS and decelerates production of peptidoglycans and LPS. *Gammaproteobacteria* with the *rpoN* operon comprising the PTS^Ntr^ genes and *rapZ* have presumably evolved the *rpoN* operon to control amino sugar homeostasis in accordance with nitrogen availability.

## Methods

### Bacterial strains, plasmids, and culture conditions

*Salmonella* was genetically manipulated using the phage λ Red recombination system^56^ and phage P22-mediated transduction^57^ with *Salmonella enterica* serovar Typhimurium SL1344 as the parent strain. All bacterial strains and plasmids used in this study are listed in Supplementary Table S1. Bacteria were grown aerobically at 37 °C in LB or M9 minimal medium supplemented with nutrients as described. Antibiotics were used at the following concentrations: ampicillin, 50 μg/ml; chloramphenicol, 25 μg/ml; and kanamycin, 50 μg/ml. A detailed description strain and plasmid construction is provided in the Supplementary information.

### Ligand-fishing to search for EIIA^Ntr^-His_6_ protein targets

*Salmonella* Typhimurium SL1344 cells were grown overnight in 300 ml LB broth at 37 °C with shaking and were harvested and resuspended in 10 ml lysis buffer [20 mM Tris-HCl (pH 8.0) and 300 mM NaCl]. The cells were disrupted by sonication on ice and then pelleted by centrifugation at 15,000 *g* for 1 h at 4 °C. The supernatant was mixed with 1 mg EIIA^Ntr^-His_6_ or not and was further incubated with 300 μl Ni-NTA resin for 1 h at 4 °C. The incubated mixture was loaded onto a Poly-Prep chromatography column (8 × 40 mm) (Bio-Rad, Hercules, CA, USA) and washed with 5 ml washing buffer [20 mM Tris-HCl (pH 8.0), 300 mM NaCl, and 5 mM imidazole], and the proteins bound to the resin were eluted with elution buffer [20 mM Tris-HCl (pH 8.0), 300 mM NaCl, and 250 mM imidazole]. Aliquots of the eluted protein samples were analyzed by SDS-PAGE and stained with Coomassie Brilliant Blue G. The protein that was specifically bound to EIIA^Ntr^-His_6_ was excised from the gel and subjected to in-gel digestion with trypsin and LC-MS/MS analysis as described previously^58^.

### Immunoprecipitation of EIIA^Ntr^ using His_6_-Glms *in vivo*

*E. coli* BL21 (DE3) cells, which produce GlmS tagged with His_6_ at its N-terminus (His_6_-GlmS) and EIIA^Ntr^ on pETDuet-*glmS*·*ptsN*, were grown in 200 ml LB, and protein expression was induced by adding 1 mM IPTG at OD_600_ of 2.0 for 6 h. The cell suspension was disrupted by sonication and centrifuged, and the supernatant was mixed with 1 ml Ni-NTA metal-affinity resin. After the mixture was loaded onto a Poly-Prep chromatography column, the column was washed four times with 2 ml washing buffer [20 mM Tris-HCl (pH 8.0), 300 mM NaCl, and 20 mM imidazole]. The proteins bound to the column were eluted with 500 μl of elution buffer [20 mM Tris-HCl (pH 8.0), 300 mM NaCl, and 50–200 mM imidazole]. Aliquots from the total cell extract (T), the supernatant after the cell extract was centrifuged (S), column flow-through (F), wash from washing (W), and the eluted fractions (E) were separated on 12% SDS-PAGE, and the gels were analyzed after staining with Coomassie Brilliant Blue G.

### Bacterial two-hybrid system for studying protein-protein interactions

The *ptsN* gene and its derivative and *glmS* gene were fused in-frame to the 3′-end of the *cyaA* gene fragments in pKT25 and pUT18C^32^,^59^, respectively, as described in the supplemental materials and methods. The *E. coli* reporter strain BTH101 was transformed with the bait and prey plasmids and cultured in LB broth containing IPTG (0.5 mM). To determine the interactions between proteins in the β-galactosidase assay, cell lysates in working buffer [70 mM Na_2_HPO_4_-H_2_O, 30 mM NaHPO_4_-H_2_O, 1 mM MgSO_4_, 0.2 mM MnSO_4_, and 100 mM β-mercaptoethanol] were incubated with o-nitrophenol-β-galactoside (4 mg/ml) as a substrate, and absorbance values at 420 nm and 550 nm were transformed into Miller units^60^.

### *In vitro* phosphorylation assay

To measure the Phosphorylation-Dependent Mobility Shift (PDMS) of EIIA^Ntr^, EIIA^Ntr^-His_6_ (K75D) (2 μg) was incubated with His_6_-EI^Ntr^ (1 μg) and His_6_-NPr (1 μg) in the presence or absence of PEP (1 mM) in a total volume of 20 μl containing 0.1 M Tris-HCl (pH 7.5), 2 mM MgCl_2_, 1 mM EDTA, 10 mM KCl, and 0.5 mM dithiothreitol^22^. After a 20 min incubation at 37 °C, the reaction was stopped by adding 5 μl 4× SDS-Sample Buffer (L1100-001; GeneDEPOT, Barker, TX, USA) [250 mM Tris-HCl (pH 6.8), 40% glycerol, 8% SDS, and 8% β-mercaptoethanol], and the aliquots were analyzed by 15% SDS-PAGE. The proteins were stained with Coomassie Brilliant Blue G. To examine the effect of various metabolites on PTS^Ntr^ phosphotransferase activity, EIIA^Ntr^-His_6_ (K75D) (3 μg) was incubated with PEP (1 mM), His_6_-EI^Ntr^ (0.3 μg), and His_6_-NPr (0.3 μg) in the presence of Gln, α-KG, or GlcN6P at 5 mM each. After a 5 min incubation at 37 °C, the reactions were stopped and processed as described above.

### *In vitro* protein-protein interactions between phosphorylated/unphosphorylated EIIA^Ntr^ and GlmS

To compare the binding affinities of EIIA^Ntr^ to GlmS depending on phosphorylation status, EIIA^Ntr^-His_6_ (K75D) (100 μg) was incubated with His_6_-EI^Ntr^ (10 μg) and His_6_-NPr (10 μg) in the presence or absence of PEP (2 mM) in a total volume of 100 μl. PEP-dependent phosphorylation of EIIA^Ntr^-His_6_ (K75D) was verified by 15% SDS-PAGE analysis as described above, and the remainder (95 μl of each) that was not used in EIIA^Ntr^-His_6_ (K75D) PDMS was mixed with Ni-NTA resin (50 μl) in 1 ml binding buffer [20 mM Tris-HCl (pH 8.0), and 300 mM NaCl] and incubated at 4 °C for 30 min. Purified GlmS (100 μg) was cleaved by thrombin to detach the His_6_-tag from His_6_-GlmS and was added to each reaction and incubate for 30 min at 4 °C under the same conditions. To dissociate the bound proteins, elution buffer [20 mM Tris-HCl (pH 8.0), 300 mM NaCl, and 250 mM imidazole] was passed through the Ni-NTA resin several times. The eluent was subsequently analyzed by SDS-PAGE, followed by Coomassie Brilliant Blue staining.

### Size exclusion chromatography with multi-angle light scattering

SEC-MALS experiments were performed using a fast protein liquid chromatography system (GE Healthcare) connected to a Wyatt MiniDAWN TREOS MALS instrument and a Wyatt Optilab rEX differential refractometer. A Superdex 200 10/300 GL (GE Healthcare) gel-filtration column pre-equilibrated with equilibrium buffer [20 mM Tris-HCl (pH 8.0) and 300 mM NaCl] was normalized using ovalbumin protein. Proteins (2 mg) were injected at a flow rate of 0.5 ml/min. Data were analyzed using the Zimm model for static light-scattering data fitting and graphed using EASI graph with a UV peak in ASTRA V software (Wyatt Technology Corp., Goleta, CA, USA).

### Glucosamine-6-phosphate synthase activity assay

The following stock solutions were prepared before the GlmS activity assay: 0.5 M Tris-HCl (pH 7.5), 0.5 M KCl, 10 mM EDTA (pH 7.5), 60 mM Fru6P, and 50 mM L-Gln. A pre-warmed solution containing 6 mM Fru6P, 10 mM L-Gln in 50 mM Tris-HCl (pH 7.5), 50 mM KCl, and 1 mM EDTA was mixed with GlmS (0–16 pmol) and incubated at 37 °C for 30 min. GlmS activity was inactivated by heating at 80 °C for 20 min, and the GlcN6P product was measured by HPLC. To determine the effects of EIIA^Ntr^ on GlmS activity, different amounts of EIIA^Ntr^-His_6_ (K75D) (0–16 pmol) were added to the reaction, and the levels of GlcN6P produced were compared. Reaction mixtures without GlmS or Fru6P were used as negative controls. A detailed description of the procedure can be found in ref. 61.

### RNA isolation and quantitative real-time RT-PCR

*Salmonella* strains were grown in LB or W-salts medium^24^. W-salts medium was supplemented with 20 mM alanine and 0.26 mM histidine. Different carbon sources were added separately when required: 0.2% GlcNAc, 0.2% glucose or 0.2% glycerol. Total RNAs were isolated at the mid-log phase using the RNeasy mini kit (Qiagen, Valencia, CA, USA). After DNase treatment of the isolated total RNAs, cDNA was synthesized with the RNA to cDNA EcoDry^TM^ Premix and random hexamers (Clontech, Palo Alto, CA, USA). The synthesized cDNA was mixed with 2 × iQ SYBR Green Supermix (Bio-Rad), and real-time PCR was performed using the CFX 3.1 (Bio-Rad). mRNA expression levels of target genes were normalized relative to that of the *gyrB* (DNA gyrase subunit B). All qRT-PCR primer sets used in this study are listed in the Supplementary Table S4.

### SDS-PAGE and Western blotting analysis

Protein samples (purified or interaction complexes) or whole-cell fractions (cell extract after sonication or cell pellets) were dissolved in Laemmli sample buffer and boiled for 5 min. The protein samples loaded onto 12% or 15% SDS-PAGE gels were separated based on molecular weights, and transferred to a polyvinylidene difluoride membrane. The membrane was blocked with 5% nonfat dry milk in 1× Tris-buffered saline-Tween 20 (TBS-T) buffer and probed with anti-FLAG antibody (3:2,000 dilution, F1804; Sigma, St. Louis, MO, USA), anti-His_6_ antibody (3:2,000 dilution, sc-8036; Santa Cruz Biotechnology, Santa Cruz, CA, USA), anti-DnaK antibody (1:10,000 dilution, ADI-SPA-880; Enzo Life Science, Farmingdale, NY, USA) or anti-RpoB antibody (1:10,000 dilution, sc-56766; Santa Cruz Biotechnology, Santa Cruz, CA, USA) as primary antibodies. Anti-mouse IgG conjugated with peroxidase (3:5,000 dilution, sc-2005; Santa Cruz Biotechnology) was used as the secondary antibody in all Western blots. The chemiluminescent signals were developed with a West-Zol plus Western blot detection system (Intron Biotechnology, Seoul, South Korea).

## Additional Information

**How to cite this article**: Yoo, W. *et al*. Fine-tuning of amino sugar homeostasis by EIIA^Ntr^ in *Salmonella* Typhimurium. *Sci. Rep.* **6**, 33055; doi: 10.1038/srep33055 (2016).

## Supplementary Material

Supplementary Information
