# Supplementary material for: Fine-tuning of amino sugar homeostasis by EIIANtr in Salmonella Typhimurium
Source: Sci Rep. 2016 Sep 15;6:33055. doi: 10.1038/srep33055 (PMC5024086; doi:10.1038/srep33055)
Supplement: Supplementary Information [file srep33055-s1.pdf]

Supplementary information

Fine-tuning of amino sugar homeostasis by EIIA^{Ntr} in *Salmonella* Typhimurium

Woongjae Yoo[1,†], Hyunjin Yoon[2,†], Yeong-Jae Seok[3], Chang-Ro Lee[4], Hyung Ho Lee[5], and Sangryeol Ryu[1,*]

[1]Department of Food and Animal Biotechnology, Department of Agricultural Biotechnology, Research Institute for Agriculture and Life Sciences, and Center for Food and Bioconvergence, Seoul National University, Seoul 08826, Korea

[2]Department of Molecular Science and Technology, Department of Applied Chemistry and Biological Engineering, Ajou University, Suwon 16499, Korea

[3]Department of Biological Sciences and Institute of Microbiology, Seoul National University, Seoul 08826, Korea

[4]Department of Biological Sciences, Myongji University, Yongin, Gyeonggido 17058, Republic of Korea

[5]Department of Chemistry, College of Natural Sciences, Seoul National University, Seoul 08826, Korea

*Corresponding author. Tel: +82 2 880 4856; Fax: +82 2 873 5095; E-mail: sangryu@snu.ac.kr

[†]These authors contributed equally to this work.

Materials and Methods for Supplementary information

Construction of bacterial strains

The phage λ-derived Red recombination system was used to delete genes in-frame or to fuse genes/proteins with peptide tags[1]. The Km^R cassette from pKD13 was amplified using the ptsN-del-F and ptsN-del-R, and glmS-del-F and glmS-del-R primers, respectively, to construct the SR7001 (Δ*ptsN*) and SR7002 (Δ*glmS*) strains, and the resulting PCR products were introduced into the SL1344 strain containing the pKD46 plasmid. Recombinant bacteria containing the Km^R cassette in place of the target genes were detected using kanamycin resistance and diagnostic PCR. The Km^R cassette was further removed using the pCP20 plasmid[1]. GlcNAc (0.2%) was added to the medium during Δ*glmS* selection to complement the lethality of Δ*glmS*. *lon* was deleted and $EIIA^{Ntr}$ was tagged with the FLAG peptide at the C-terminus using a phage λ-mediated recombination system[1,2]. The strain carrying a *lacZ* fusion to *ssaG* was constructed similarly as described above. The Km^R cassette that was PCR-amplified from the pKD3 plasmid using the ssaG-lacZ-F and ssaG-lacZ-R primers was introduced downstream of *ssaG* and then replaced with the *lacZY* genes of pCE70 via the FRT site. The primers used to construct the bacterial strains are listed in Supplementary Table S3.

Plasmid construction

For the construction of pWJ04 producing $EIIA^{Ntr}$ under its putative intrinsic promoter, the DNA containing the *Salmonella ptsN* gene was amplified by PCR using primers of ptsN-comple-F and ptsN-comple-R, and the PCR products were introduced between the HindIII and SphI sites of pACYC184. To construct pWJ06 expressing $EIIA^{Ntr}$ under the *lac* promoter, the *ptsN* gene was PCR amplified using the ptsN-F and ptsN-R primers and the purified PCR

fragments were inserted between the EcoRI and BamHI sites of the pUHE21-2*lacI*^{q} vector[3]. The *ptsN* gene was also amplified using the ptsN-His-F and ptsN-His-R primers, and the PCR products were introduced between the EcoRI and BamHI sites of the pUHE21-2*lacI*^{q} vector to construct pWJ07 producing $EIIA^{Ntr}$ tagged with six histidines at its C-terminus. The pWJ10 plasmid expressing GlmS from the *lac* promoter was constructed by introducing PCR fragments containing *glmS* between the BamHI and HindIII sites of the pUHE21-2*lacI*^{q} vector. The glmS-F and glmS-R primers were used to construct pWJ10. The pKT25-*ptsN* and pUT18C-*glmS* plasmids were constructed for the bacterial two-hybrid analysis. The *ptsN* and *glmS* genes were amplified using the ptsN-BTH-F and ptsN-BTH-R, and glmS-BTH-F and glmS-BTH-R primers, respectively. The purified PCR products were inserted between the BamHI and EcoRI sites of the pKT25 plasmid vector or between the BamHI and SacI sites of the pUT18C plasmid vector[4]. The pWJ11, pWJ12, pWJ13, and pWJ14 plasmids were constructed to extract and purify EI^{Ntr}, NPr, $EIIA^{Glc}$, and GlmS proteins, respectively. The pET28a vector was used to purify soluble proteins containing a His_6-tag at their N-termini. Plasmids expressing $EIIA^{Ntr}$ with H73A and K75D substitutions were constructed using a QuickChange II site-directed mutagenesis kit (Stratagene, La Jolla, CA, USA) with the ptsN-H73A-F and ptsN-H73A-R primers for $EIIA^{Ntr}$ (H73A) and the ptsN-K75D-F and ptsN-K75D-R primers for $EIIA^{Ntr}$ (K75D). Plasmids expressing $EIIA^{Ntr}$ or its derivatives were introduced into a Δ*ptsN* mutant strain with P*ssaG-lacZ* to verify the regulatory activity of $EIIA^{Ntr}$ on *ssaG*[5], and all plasmids tested complemented the Δ*ptsN* mutant (Supplementary Fig. S1). The pWJ15, pWJ16, and pWJ17 plasmids were constructed for the pull-down assay. The pETDuet-1 vector was used to express His_6-GlmS and $EIIA^{Ntr}$. The *glmS* and *ptsN* genes were amplified using the Duet-glmS-His-F and Duet-glmS-His-R, and Duet-ptsN-F and Duet-ptsN-R primers, respectively. The purified PCR products of the *glmS* and *ptsN* genes

were inserted between the NcoI and HindIII, and NdeI and KpnI sites of the pETDuet-1 vector, respectively. The primers used to construct the plasmids in this study are listed in Supplementary Table S3.

Bacterial two-hybrid system-mediated screening. To analyze protein-protein interactions between the phosphorylated/dephosphorylated forms of EIIA^{Ntr} and GlmS *in vivo* conditions, the BACTH (Bacterial Adenylate Cyclase Two-Hybrid) system based on reconstitution of adenylate cyclase (CyaA) activity through heterodimerization of the hybrid proteins was used with the *E. coli* BTH101 reporter strain, which lacks an endogenous functional *cyaA* gene. The proteins to be tested were fused to the T25- and T18-fragments of the CyaA protein from *Bordetella pertussis*. The T25- and T18-domains are unable to interact with each other without a fusion partner. However, when fused to proteins that interact with each other, the T25- and T18- domains assemble into a functional CyaA protein that synthesizes cAMP. cAMP synthesis is monitored by the expression of cAMP-CRP-dependent genes, such as *lacZ* encoding β-galactosidase. Therefore, β-galactosidase activities reflect the strength of the interactions between the proteins fused to the CyaA domains. The pKT25 and pUT18C plasmids were used to construct the T25-CyaA and T18-CyaA fusions, respectively, to the N-termini of EIIA^{Ntr}/EIIA^{Ntr} (H73A) and GlmS as potential interaction partners. pKT25-*zip* and pUT18C-*zip* are derivatives in which the leucine zipper of GCN4 is genetically fused in frame to the T25 or T18 fragment respectively. The plasmids pKT25-*zip* and pUT18C-*zip* serve as positive controls for complementation. When pKT25-*zip* and pUT18C-*zip* are co-transformed into BTH101 strains, they restore a characteristic Cya^{+} phenotype due to the result of the dimerization of leucine zipper motifs. The *E. coli* BTH101 reporter strain was also co-transformed with two recombinant plasmids (e.g., pKT25-*ptsN* and pUT18C-*glmS*, or

pKT25-*ptsN* (H73A) and pUT18C-*glmS*) and plated on LB agar medium supplemented with kanamycin, 50 μg/ml; ampicillin, 50 μg/ml; 5-bromo-4-chloro-3-indolyl-β-D-galactoside, 40 μg/ml; and IPTG, 0.5 mM (final concentration). The plates were incubated at 30°C for 24 h or more. Blue colonies were selected and streaked on the same agar plate or inoculated into LB broth containing IPTG to validate the interactions between the tested proteins (Supplementary Fig. S2B). For the latter case, the β-galactosidase activities were determined to compare interaction strength between pairs of hybrid proteins[6,7].

Purification of His_6-tagged proteins. For purification of soluble His_6-tagged proteins (His_6-EI^{Ntr}, His_6-NPr, His_6-$EIIA^{Glc}$, and His_6-GlmS: all tagged at their N-termini; $EIIA^{Ntr}$-His_6, $EIIA^{Ntr}$-His_6 (H73A), and $EIIA^{Ntr}$-His_6 (K75D): all tagged at their C-termini), *E. coli* BL21 (DE3) or *S.* Typhimurium SL1344 Δ*glmS* strains harboring plasmids expressing His_6-tagged proteins were grown overnight in LB medium, and protein expression was induced by adding 1 mM IPTG. The His_6-tagged proteins were purified using nickel-nitrilotriacetic acid (Ni-NTA) resin (Qiagen) according to the manufacturer's instructions, and bound proteins were eluted with elution buffer [20 mM Tris-HCl (pH 8.0), 300 mM NaCl, and 250 mM imidazole] (Supplementary Fig. S11). The eluted proteins were concentrated using a VivaSpin 20 instrument (3,000-molecular-weight cutoff [MWCO] polyethersulfone; Sartorius, Göttingen, Germany), and the elution buffer was replaced with storage buffer [20 mM Tris-HCl (pH 8.0), 300 mM NaCl, and 50% glycerol] using a PD MidiTrap G-25 column (GE Healthcare, Buckinghamshire, United Kingdom). Protein concentration was determined using the Bradford assay (Bio-Rad) with BSA as the standard. To remove the His_6-tag from the purified proteins, the Thrombin Cleavage Capture Kit (69022-3; Novagen, Gibbstown, NJ, USA) was used according to the manufacturer's instructions. Cleavage of the His_6-tag from

the purified proteins was confirmed by SDS-PAGE stained with Coomassie Brilliant Blue G and Western blot analysis using anti-His_6 antibody (sc-8036; Santa Cruz Biotechnology).

Analysis of protein stability. Protein stability was determined as described previously. *Salmonella* strains producing the $EIIA^{Ntr}$-FLAG protein from the chromosome were grown in LB medium with or without 0.2% GlcNAc for 3 h, and 0.2 mg/ml chloramphenicol was added to block *de novo* protein synthesis. Aliquots of the cultures were taken at the indicated time points after adding chloramphenicol and were subjected to Western blotting, as described above.

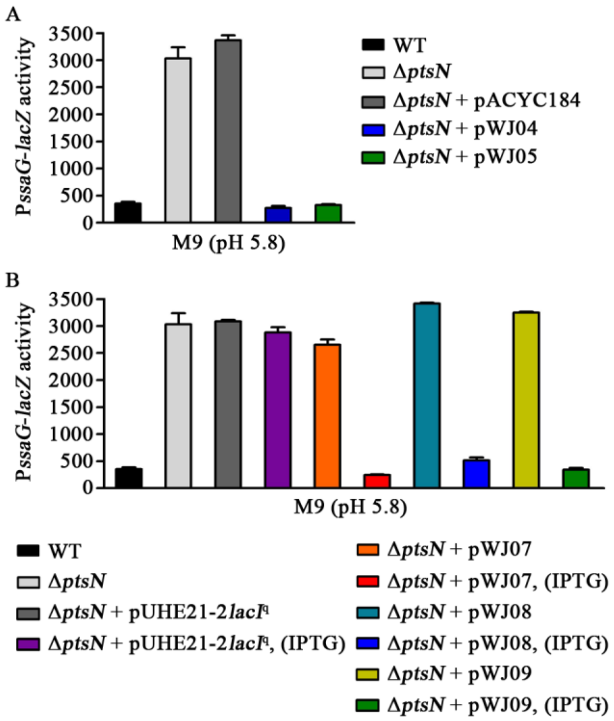

Figure S1. EIIA^{Ntr} derivatives constructed in this study did not impair the regulatory role of EIIA^{Ntr} in *Salmonella* virulence gene expression. (A) Evaluation of the regulatory activities of EIIA^{Ntr} and its derivative EIIA^{Ntr} (H73A) for controlling *ssaG* expression. Intrinsic EIIA^{Ntr} negatively regulates *ssaG*, a component gene of *Salmonella* pathogenicity island 2 (SPI-2)[5]. A Δ*ptsN* mutant strain containing P*ssaG-lacZ* was transformed with pWJ04 and pWJ05, respectively, in parallel with pACYC184 as a backbone. The plasmids of pWJ04 and pWJ05 were designed to produce EIIA^{Ntr} and its derivative EIIA^{Ntr} (H73A), respectively, in *trans*. *Salmonella* strains were cultivated in M9 minimal medium (pH 5.8) for 8 h, and *ssaG* expression was compared in triplicate using β-galactosidase assay. **(**B) Evaluation of the regulatory activities of His$_6$-tagged EIIA^{Ntr} and its derivatives for controlling *ssaG* expression. Plasmids of pWJ07, pWJ08, and pWJ09 using pUHE21-2*lacI*^{q} as a backbone were introduced into the Δ*ptsN* mutant strain harboring P*ssaG-lacZ*. They were designed to express EIIA^{Ntr}-His$_6$, EIIA^{Ntr}-His$_6$ (H73A), and EIIA^{Ntr}-His$_6$ (K75D), respectively, upon induction with IPTG (0.1 mM). The β-galactosidase assay was performed in triplicate as described

above in the presence or absence of IPTG (0.1 mM).

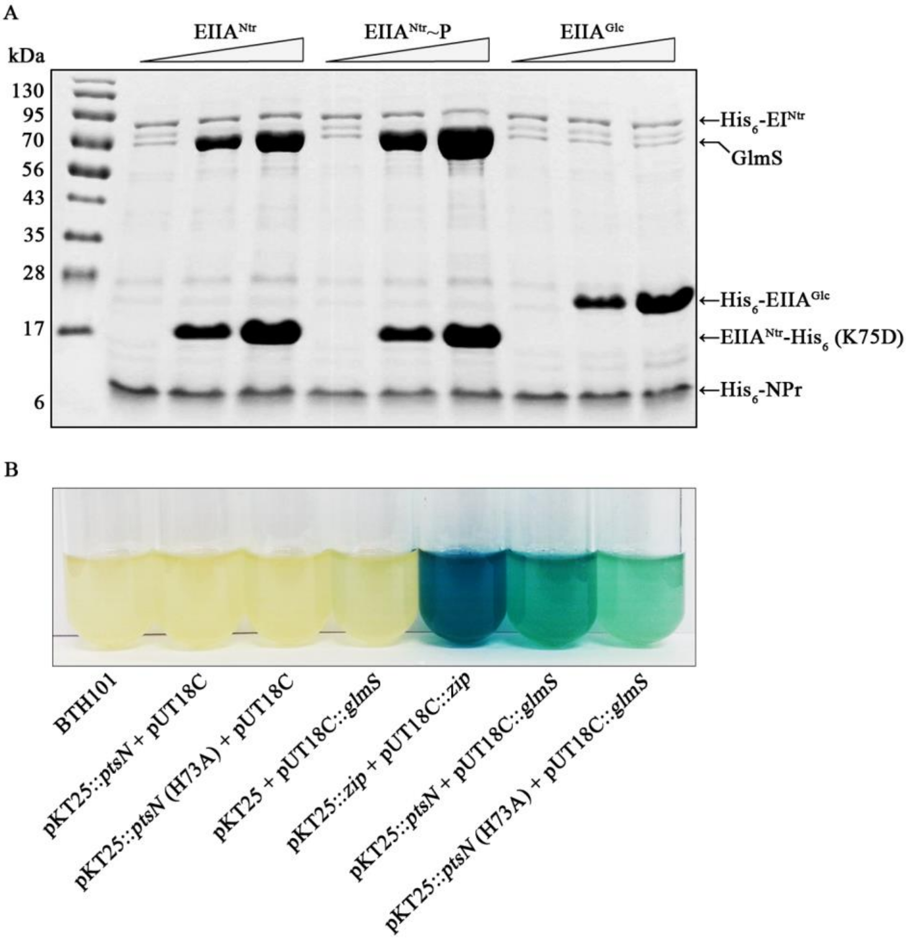

Figure S2. EIIA^{Ntr} binds to GlmS directly in a phosphorylation-status-dependent manner. (A) Phosphorylation and concentration-dependent interactions between EIIA^{Ntr} and GlmS. GlmS (100 µg) was incubated with various amounts (0, 25, and 100 µg) of EIIA^{Ntr}-His$_6$ (K75D) or His$_6$-EIIA^{Glc} and subjected to a pull-down assay using Ni-NTA metal affinity resin. EIIA^{Ntr}-His$_6$ (K75D) was phosphorylated or not depending on the presence of PEP. (B) Increase in the interactions between EIIA^{Ntr} and GlmS by its phosphorylation *in vivo*. The pKT25 plasmid containing *ptsN*, *ptsN* (H73A) or *zip* and the pUT18C plasmid harboring *glmS* or *zip* were introduced into a reporter *E. coli* strain individually or in combination. All strains were grown in LB medium containing X-gal, and colorimetric X-gal degradation was compared.

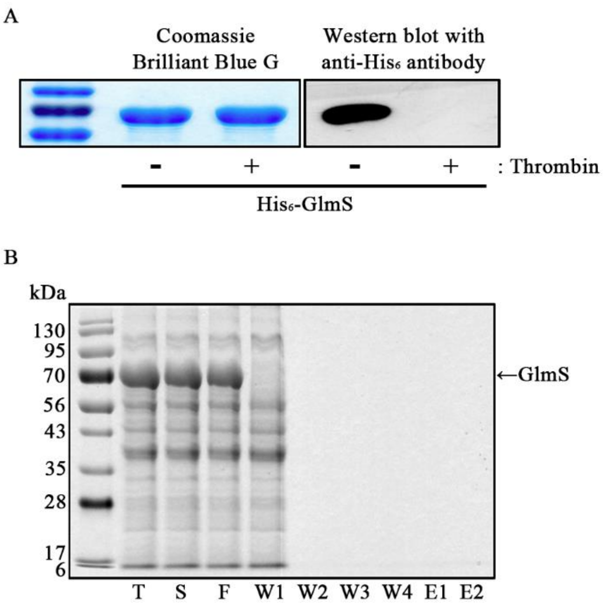

Figure S3. GlmS does not bind to anti-His$_6$ antibody and Ni-NTA resin non-specifically. (A) Purified His$_6$-GlmS proteins were incubated at 37 °C for 6 h in the presence or absence of thrombin protease to detach His$_6$-tag from GlmS. Samples treated with thrombin or not were separated on 12% SDS-PAGE gel. The protein gel was stained with Coomassie Brilliant Blue G, or further analyzed by western blot using anti-His$_6$ antibody. (B) Bacteria containing pWJ10 (pUHE21-2*lacI*^{q}::*glmS*) over-produced GlmS protein upon IPTG addition and their lysates were incubated with Ni-NTA affinity resin to examine non-specific binding of GlmS to the resin. Aliquots from total cell extracts (T), supernatant after centrifugation of the cell extracts (S), column flow-through (F), washes from four washing steps (W), and the two eluted fractions (E) were separated on 12% SDS-PAGE, and the gels were analyzed after Coomassie Brilliant Blue G staining. Molecular masses of the standards are presented in kDa on the left.

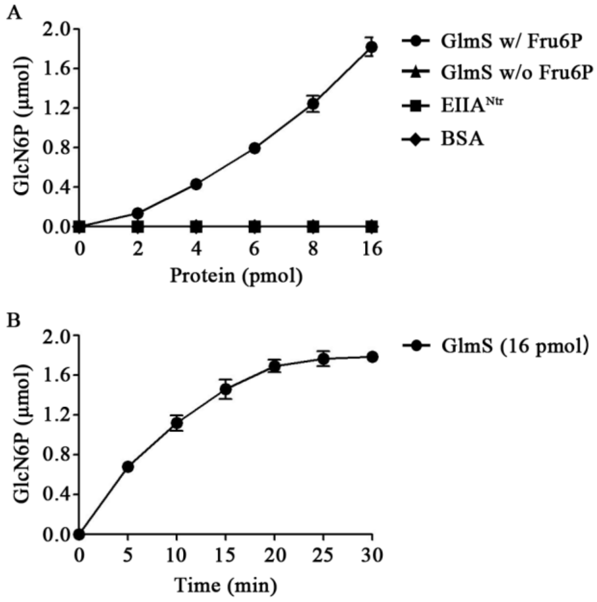

Figure S4. A method to assay GlmS activity was designed by measuring GlcN6P using HPLC. (A) GlcN6P production at different GlmS concentrations. Gln (10 mM) was mixed with GlmS (0–16 pmol) in the presence or absence of Fru6P (6 mM) and incubated at 37℃ for 30 min. GlcN6P production increased in proportion to the quantity of enzyme added. $EIIA^{Ntr}$-His_6 (K75D) and BSA were used as negative controls. (B) GlcN6P production using different reaction times. To optimize incubation time, GlcN6P was measured every 5 min after adding 16 pmol GlmS to the reaction mixture containing Gln and Fru6P. Maximal GlcN6P production was observed at 30 min.

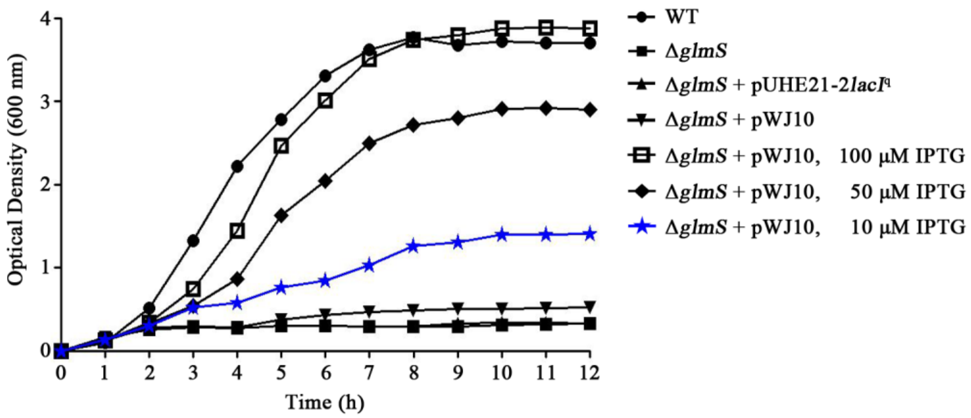

Figure S5. Lethality due to the absence of GlmS was modulated by GlmS production in *trans*. *Salmonella* was cultured at 37℃ in LB medium for 12 h to compare growth rate between strains. A Δ*glmS* mutant strain with a growth defect was supplemented by the introduction of pWJ10 (a pUHE21-2*lacI*^{q} derivative harboring *glmS* under a *lac* promoter) under different IPTG concentrations (10–100 μM). Bacterial growth rates were compared at 37℃ in LB medium for 12 h. The 10 μM IPTG concentration showing partial growth complementation was chosen for further study. This experiment was performed in triplicate.

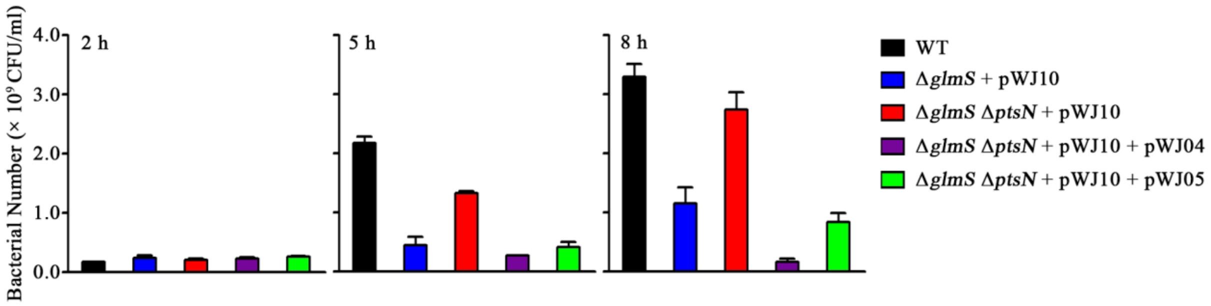

Figure S6. Viability of *Salmonella* is influenced by the interaction between GlmS and EIIA^{Ntr}. To compare the bacterial viability among *Salmonella* strains wild-type, Δ*glmS*, and Δ*glmS* Δ*ptsN* strains containing pWJ10 (pUHE21-2*lacI*^{q}::*glmS*), pWJ04 (pACYC184::*ptsN*), or pWJ05 (pACYC184::*ptsN*_H73A), culture aliquots were collected at 2, 5, and 8 h time points and then serially diluted in PBS. Dilutions of the suspension were plated on LB agar medium to enumerate the CFU. This experiment was performed in triplicate.

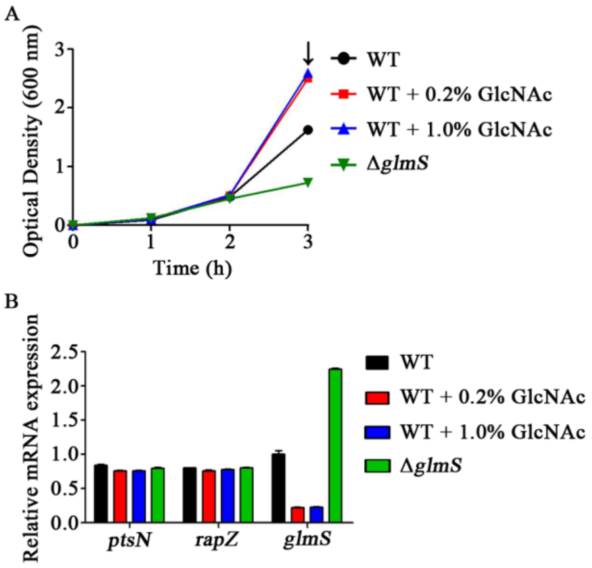

Figure S7. *ptsN* and *rapZ* mRNA expression was not influenced by the abundance of amino sugars. (A) Estimate of growth time when amino sugar availability may influence bacterial growth. Wild-type and Δ*glmS* mutant strains were cultured in LB medium supplemented with GlcNAc (0.2 or 1.0%) or not. *Salmonella* cells began to show different growth rates after 3 h, depending on the presence of GlmS or availability of GlcNAc, suggesting that intracellular amino sugars were depleted at around 3 h and the growth got attenuated unless GlmS synthesized GlcN6P or exogenous GlcNAc was provided. (B) Effect of amino sugar abundance on *ptsN*, *rapZ*, and *glmS* mRNA levels. Total RNAs were isolated from wild-type and Δ*glmS* mutant strains in the presence or absence of GlcNAc at 3 h under the conditions described above and subjected to qRT-PCR. *ptsN* and *rapZ* were expressed equivalently between the conditions, whereas *glmS* expression was significantly influenced by the availability of amino sugar. Gene expression was normalized based on *gyrB* expression. qRT-PCR was performed in triplicate.

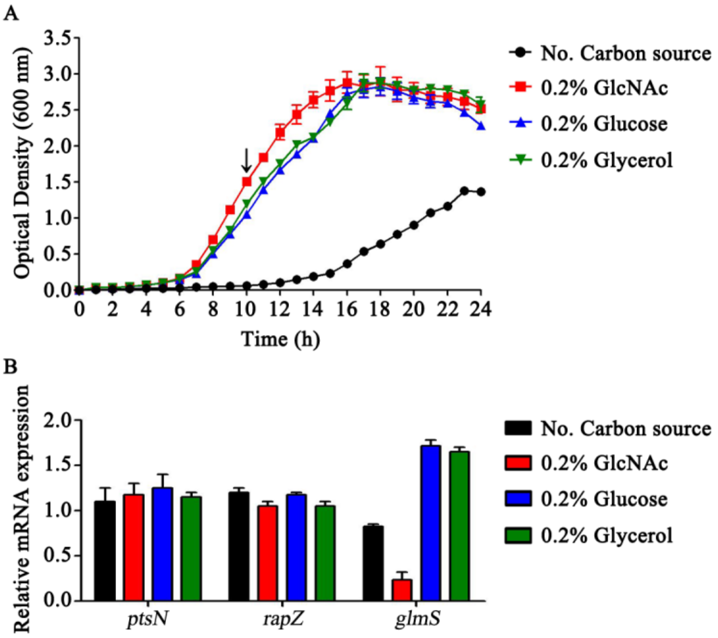

Figure S8. *ptsN* and *rapZ* mRNA expression was independent of carbon source. (A) *Salmonella* Typhimurium growth curves with different carbon sources. Wild-type *Salmonella* was cultured in minimal medium (W-salts) containing either GlcNAc, glucose, or glycerol, respectively. Bacterial growth was depicted, and total RNAs were isolated 10 h post-inoculation. (B) Comparison of *ptsN*, *rapZ*, and *glmS* expression levels in response to the different carbon sources. mRNA levels were compared between the different carbon sources using qRT-PCR. *gyrB* expression was used to normalize gene expression between tested conditions. Relative mRNA expression normalized using *gyrB* mRNA was averaged from three independent qRT-PCR tests.

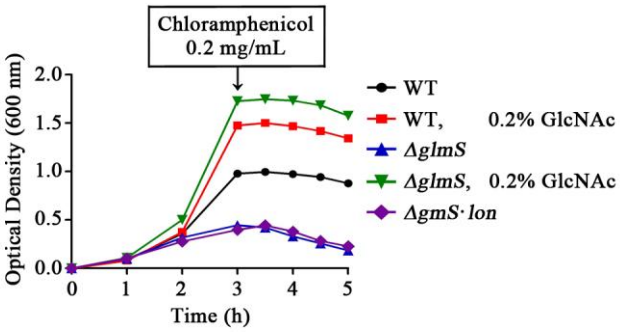

Figure S9. *Salmonella* growth rate is affected by amino sugar availability. Wild-type, Δ*glmS*, and Δ*glmS* Δ*lon* strains used in Fig. 7 were cultured in the presence or absence of GlcNAc (0.2%), and chloramphenicol (0.2 mg/ml) was added to the culture 3 h after inoculation to quench further protein synthesis. The availability of amino sugar began to affect bacterial growth rate at around 3 h, which was chosen to determine the EIIA^{Ntr}-FLAG levels under each condition.

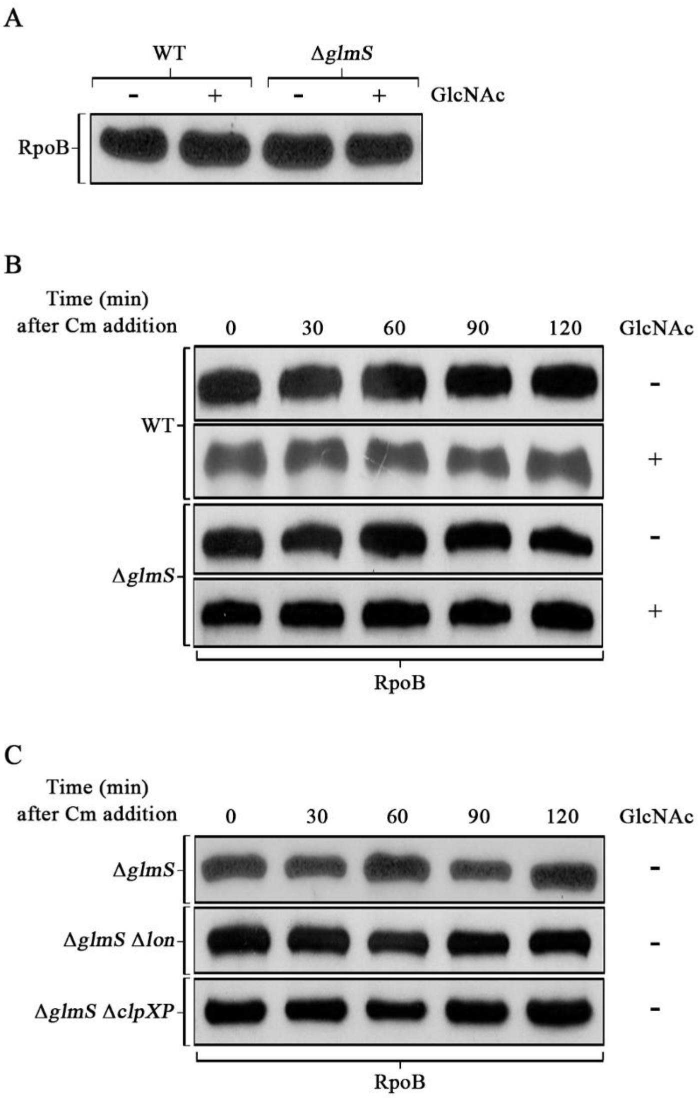

Figure S10. The stability of RpoB was not changed by Lon protease. The identical protein samples used in Fig. 7 were applied in western blot analysis using anti-RpoB antibody. The RpoB levels were comparable between lanes regardless of the presence of Lon and ClpXP.

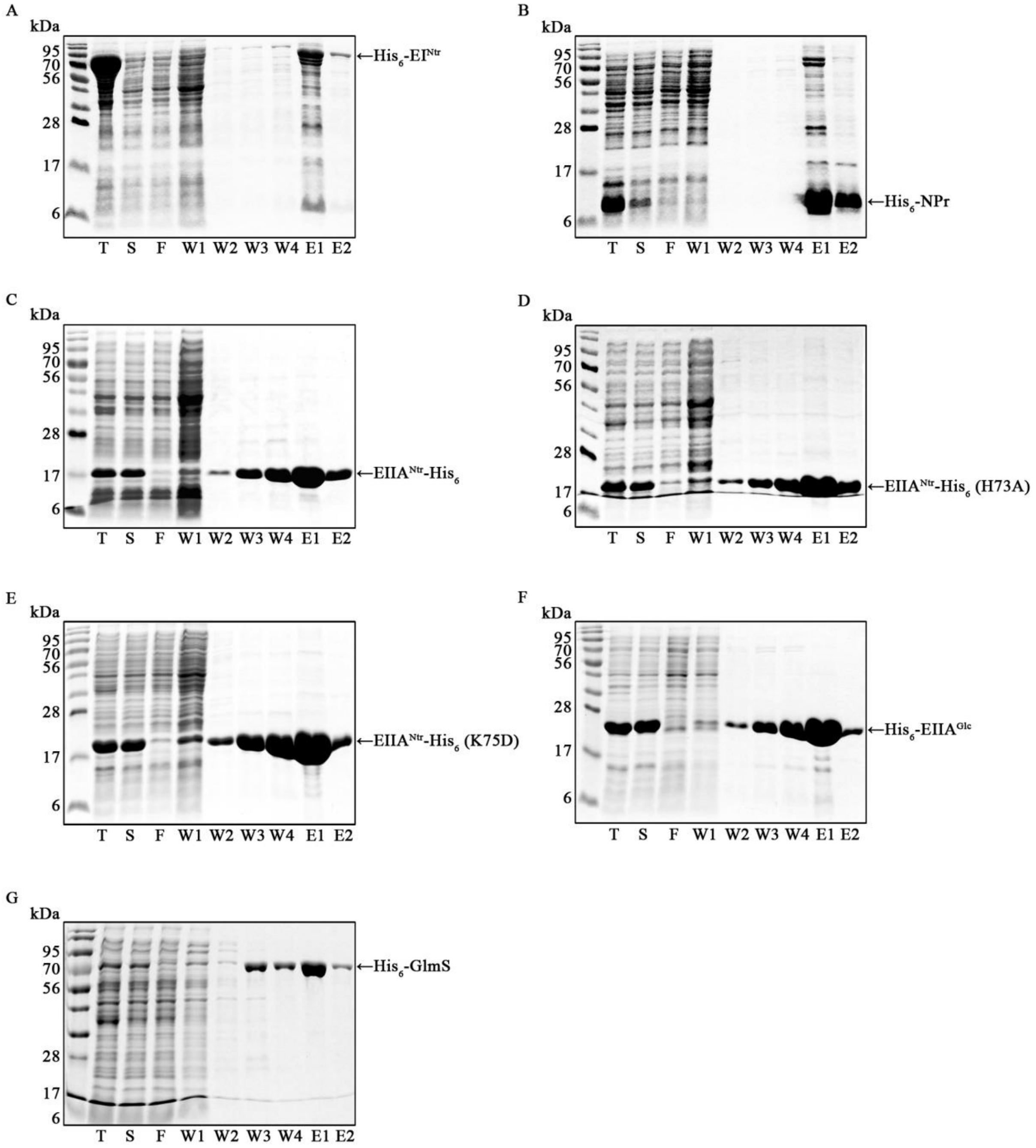

Figure S11. Over-expressed proteins were purified using affinity chromatography. SDS-PAGE analyses to examine the purity of isolated proteins. Bacterial transformants over-producing His$_6$-tagged proteins after adding IPTG were lysed, and the lysates were subjected to Ni-NTA affinity purification. Aliquots from total cell extracts (T), supernatant after centrifugation of the cell extracts (S), column flow-through (F), washes from four washing

steps, and the two eluted fractions were separated on 12% SDS-PAGE, and the gels were analyzed after Coomassie Brilliant Blue G staining. Figures of (A) to (G) denote purification of His_6-EI^{Ntr}, His_6-NPr, $EIIA^{Ntr}$-His_6, $EIIA^{Ntr}$-His_6 (H73A), $EIIA^{Ntr}$-His_6 (K75D), His_6-$EIIA^{Glc}$, and His_6-GlmS, respectively. Molecular masses of the standards are presented in kDa on the left.

Table S1. Bacterial strains and plasmids used in this study.		
**Strains**	**Description**	**Reference or source**
***Salmonella enterica* serovar Typhimurium**		
SL1344	Wild type, Sm^R	8
SR7001	Δ*ptsN*	This study
SR7002	Δ*glmS*	This study
SR7003	Δ*glmS* Δ*ptsN*	This study
SR7011	P*ssaG-lacZ*	This study
SR7012	P*ssaG-lacZ*, Δ*ptsN*	This study
SR7021	$EIIA^{Ntr}$-FLAG	This study
SR7022	$EIIA^{Ntr}$-FLAG, Δ*glmS*	This study
SR7023	$EIIA^{Ntr}$-FLAG, Δ*glmS* Δ*lon*	This study
SR7024	$EIIA^{Ntr}$-FLAG, Δ*glmS* Δ*clpXP*	This study
Escherichia coli		
BTH101	F^- *cyaA-99 araD139 galE15 galK16 rpsL1 hsdR* ^{2} μ*rA1* μ*rB1*	9
DH5α	*gyrA96 recA1 relA1 endA1 thi-1 hsdR17 glnV44 deoR Δ(lacZYA-argF)U169* [Φ80d Δ(*lacZ*)*M15*]	10
BL21 (DE3)	F^- *ompT* $hsdS_B$ (r_B^- m_B^-) *gal dcm* (DE3)	11
**Plasmids**		
pKD46	Ap^R P_{BAD}-*gam-beta-exo oriR101* $repA101^{ts}$	1
pKD13	Ap^R FRT Km^R FRT PS1 PS4 *oriR6K*γ	1

pCP20	Ap^R Cm^R *cI*857 λP_R*flp oripSC101*^{ts}	1
pCE70	Km^R FRT *tnpR lacZY*$^+$ *oriR6Kγ*	12
pACYC184	Tet^R Cm^R p15A *ori*	13
pUHE21-2*lacI*^{q}	rep_{pMB1} Ap^R *lacI*^{q}	3
pKT25	ori p15A, *Plac*::*cyaA* 1–224, MCS, *neo*	4
pUT18C	ColEI-ori, *Plac*::*cyaA* 225–399, MCS, *bla*	4
pKT25-zip	ori p15A, *Plac*::*cyaA* 1–224ΦGCN4-zip, *neo*	4
pUT18C-zip	ColEI-ori, *Plac*::*cyaA* 225–399ΦGCN4-zip, *bla*	4
pET28a	Expression vector with a hexahistidine tag, Kan^r	Novagen
pETDuet-1	Co-expression vector with a hexahistidine tag, Amp^r	Novagen
pWJ01	pKT25-*ptsN*	This study
pWJ02	pKT25-*ptsN* (H73A)	This study
pWJ03	pUT18C-*glmS*	This study
pWJ04	pACYC184-*ptsN*	This study
pWJ05	pACYC184-*ptsN* (H73A)	This study
pWJ06	pUHE21-2*lacI*^{q}-*ptsN*	This study
pWJ07	pUHE21-2*lacI*^{q}-*ptsN*-His_6	This study
pWJ08	pUHE21-2*lacI*^{q}-*ptsN*-His_6 (H73A)	This study
pWJ09	pUHE21-2*lacI*^{q}-*ptsN*-His_6 (K75D)	This study
pWJ10	pUHE21-2*lacI*^{q}-*glmS*	This study
pWJ11	pET28a-*ptsP*	This study

pWJ12	pET28a-*ptsO*	This study
pWJ13	pET28a-*crr*	This study
pWJ14	pET28a-*glmS*	This study
pWJ15	pETDuet-*glmS*	This study
pWJ16	pETDuet-*ptsN*	This study
pWJ17	pETDuet-*glmS-ptsN*	This study

Table S2. Proteins identified in the ligand fishing-associated LC-MS/MS.		
**Gene**	**Locus_tag**	**Function**
tlpA	SL1344_P1_0059	Alpha-helical coiled coil protein
yjbF	SL1344_4159	Hypothetical lipoprotein
polA	SL1344_3947	Reversed DNA polymerase I
kdpB	SL1344_0687	Reversed K^+-transporting ATPase B chain
tufB	SL1344_4085	Elongation factor Tu
groEL	SL1344_4267	60 kDa chaperonin
	SL1344_3498	Hypothetical dehydratase
glmS	SL1344_3828	Glutamine--fructose-6-phosphate aminotransferase

**Table S3. Primers used to construct the bacterial strains and plasmids.**	
**Primers**	**Sequences (5' to 3')**
ptsN-del-F	CTG GCC ATC AAC CTG ACA GGA CAG GTT CTT AGG TGA AAT TTG TAG GCT GGA GCT GCT TCG
ptsN-del-R	CGT TTC GCC ACC AGC GAC AGC GTG TGC AGA TGC GTT TTA ATT CCG GGG CTC CGT CGA CC
glmS-del-F	TTG CGC TCG AAG GCG CGC TGA AGC TGA AAG AGA TCT CTT ATG TAG GCT GGA GCT GCT TCG
glmS-del-R	CG TCT GTC GAC GGC CTT CTG CCT GGT ACT ACA TTT GTA CAT TCC GGG GAT CCG TCG ACC
lon-del-F	ATC TGA TTA CCT GGC GGA CAC TAA ACT AAG AGA GAG CTC TTG TAG GCT GGA GCT GCT TCG
lon-del-R	GCG CAC GCA CCA CGG TCA GCG CCG CCT GAA TGG ATT CCT GAT TCC GGG GAT CCG TCG ACC
clpXP-del-F	AGT ACA GCA GAT TTT TTC AAT TTT TAT CCA GGA GAC GGA ATG TAG GCT GGA GCT GCT TCG
clpXP-del-R	ACT GCT TGG TCA GCG CAT TTT TCG GCT CTT TCA GGA TTT GAT TCC GGG GAT CCG TCG ACC
ptsN-FLAG-F	TCA AAT CAT TAC TGA CAC CGA AGG TGA GCA GAA TGA GGC A GGC AGC GGC GAC TAC AAA GAC GAT GAC GAC AAG TAA TGT AGG CTG GAG CTG CTT CG
ptsN-FLAG-R	GTT TCT CCT CAC AAC GAC AGA AAT AAA TGC CAT TGA GTT GAT TCC GGG GAT CCG TCG ACC
ssaG-lacZ-F	ATA TTT ATT AAT TAC GAA AGT TCA CTG ATC GTG TAG GCT

	GGA GCT GCT TC
ssaG-lacZ-R	AAT AAA ATT TTC GCG GCT TTT AGC GGC TCA ATG GGA ATT AGC CAT GGT CC
ptsN-comple-F	AAA AAG CTT ACG CAC ATC TCG GAT GCG AC
ptsN-comple-R	AAA GCA TGC CCG CTG ACG ATC ATC AGT AC
ptsN-F	CAG GTT CTG AAT TCA AAT TAT GA
ptsN-R	GTA CCA GGA TCC GTT TCT C
ptsN-His-F	AAA GGA TCC ATG ATA AAT AAC GAT ACG AC
ptsN-His-R	AAA AAG CTT TCA GTG GTG GTG GTG GTG GTG GCC GCT GCC TGC CTC ATT CTG CTC ACC TT
ptsN-BTH-F	AAA GGA TCC TAT GAT AAA TAA CGA TAC GA
ptsN-BTH-R	AAA GAA TTC ACA ACG ACA GAA ATA AAT GC
ptsN-H73A-F	GTA ATG GTA TCG CCA TCC CGG CGG GGA AAC TGG AAG AAG ATA C
ptsN-H73A-R	GTA TCT TCT TCC AGT TTC CCC GCC GGG ATG GCG ATA CCA TTA C
ptsN-K75D-F	GCC ATC CCG CAC GGG GAT CTG GAA GAA GAT ACC T
ptsN-K75D-R	AGG TAT CTT CTT CCA GAT CCC CGT GCG GGA TGG C
glmS-F	AAA GGA TCC ATG TGT GGA ATT GTT GGC GC<br>AAA AAG CTT GCT CTT CAG CCA CCA TAG AG
glmS-R	AAA AAG CTT GCT CTT CAG CCA CCA TAG AG
glmS-BTH-F	AAA GGA TCC TAT GTG TGG AAT TGT TGG C
glmS-BTH-R	AAA GAG CTC CTT CTG CCT GGT ACT ACA TT

ptsP-His-F	AAA CAT ATG CTC ACT CGC CTG CGC
ptsP-His-R	AAA GAG CTC TGT TGT CTG CGC CGT GAA TC
ptsO-His-F	AAA CAT ATG ACC GTA AAG CAA ACT GTT G
ptsO-His-R	AAA GAG CTC GAC TGT CAT CAA ATA CCG GG
crr-His-F	AAA CAT ATG GGT TTG TTC GAT AAA CTA AA
crr-His-R	AAA GAG CTC CAC TGC GGC AAG AAT TAC TT
glmS-His-F	AAA CCA TGG CTA TGT GTG GAA TTG TTG GCG
glmS-His-R	AAA CTC GAG CTC TAC GGT AAC CGA TTT CGC
Duet-glmS-His-F	AAA CCA TGG CTA TGT GTG GAA TTG TTG GC
Duet-glmS-His-R	AAA AAG CTT CTT CTG CCT GGT ACT ACA TT
Duet-ptsN-F	AAA CAT ATG ATA AAT AAC GAT ACG AC
Duet-ptsN-R	AAA GGT ACC ACA ACG ACA GAA ATA AAT GC

Table S4. qRT-PCR primers.

**Primers**	**Sequences (5’ to 3’)**
qRT-glmS-F1	CTG AAA CCG ACA CCG AAG TG
qRT-glmS-R1	CAG AAC AGC CTC ACG CAG AGT
qRT-glmU-F1	GCG CGG ACA TGA AAC GTT
qRT-glmU-R1	CTG TTC GGC CTG GTA AAT ACG
qRT-glmM-F1	GCC TCG TTT ACC GGA CCT ATG
qRT-glmM-R1	AGG CAG TTT CGT ACC GTC GAT
qRT-ptsN-F1	AGC GGC GTT CAT TGT CAG A
qRT-ptsN-R1	GGA GGT AAA CTG AGC TGT TTT GC
qRT-rapZ-F1	GAT GAA TAA TCT GCC TGG TGC TT
qRT-rapZ-R1	TGT AGC GGC GAA TCA AGG TA
qRT-gyrB-F1	ATA ACG CCA CGC AGA AAA TGA
qRT-gyrB-R1	TGG CTG ATA CAC CAG CTC TTT G

References

1. Datsenko, K.A. & Wanner, B.L. One-step inactivation of chromosomal genes in *Escherichia coli* K-12 using PCR products. *Proc. Natl. Acad. Sci. USA* **97,** 6640-6645 (2000).
2. Ellermeier, C.D., Janakiraman, A. & Slauch, J.M. Construction of targeted single copy *lac* fusions using λ RED and FLP-mediated site-specific recombination in bacteria. *Gene* **290,** 153-161 (2002).
3. Soncini, F.C., Vèscovi, E.G. & Groisman, E.A. Transcriptional autoregulation of the *Salmonella typhimurium phoPQ* operon. *J. Bacteriol.* **177,** 4364-4371 (1995).
4. Karimova, G., Ullmann, A. & Ladant, D. Protein-protein interaction between *Bacillus stearothermophilus* tyrosyl-tRNA synthetase subdomains revealed by a bacterial two-hybrid system. *J. Mol. Microbiol. Biotechnol.* **3,** 73-82 (2001).
5. Choi, J. *et al*. *Salmonella* pathogenicity island 2 expression negatively controlled by $EIIA^{Ntr}$-SsrB interaction is required for *Salmonella* virulence. *Proc. Natl. Acad. Sci. USA* **107,** 20506-20511 (2010).
6. Miller, J.H. Experiments in molecular genetics (Cold Spring Harbor Laboratory Press, Plainview, NY) (1972).
7. Karimova, G., Pidoux, J., Ullmann, A. & Ladant, D. A bacterial two-hybrid system based on a reconstituted signal transduction pathway. *Proc. Natl. Acad. Sci. USA* **95,** 5752-5756 (1998).
8. Robin, L. Lucas. & Lee, C.A. Unravelling the mysteries of virulence gene regulation in *Salmonella typhimurium*. *Mol. Microbiol.* **36,** 1024-1033 (2000).
9. Karimova, G., Ullmann, A. & Ladant, D. A bacterial two-hybrid system that exploits a cAMP signaling cascade in *Escherichia coli*. *Methods. Enzymol.* **328,** 59-73 (2000).

10. Woodcock, D.M. *et al*. Quantitative evaluation of *Escherichia coli* host strains for tolerance to cytosine methylation in plasmid and phage recombinants. *Nucleic. Acids. Res.* **17,** 3469-3478 (1989).

11. Studier, F.W. & Moffattf, B.A. Use of bacteriophage T7 RNA polymerase to direct selective high-level expression of cloned genes. *J. Mol. Biol.* **189,** 113-130 (1986).

12. Merighi, M., Ellermeier, C.D., Slauch, J.M. & Gunn, J.S. Resolvase-in vivo expression technology analysis of the *Salmonella enterica* serovar Typhimurium PhoP and PmrA regulons in BALB/c mice. *J. Bacteriol.* **187,** 7407-7416 (2005).

13. Chang, A.C.Y. & Cohen, S.N. Construction and characterization of amplifiable multicopy DNA cloning vehicles derived from the P15A cryptic miniplasmid. *J. Bacteriol.* **134,** 1141-1156 (1978).
